# Gasdermin D in peripheral myeloid cells drives neuroinflammation in experimental autoimmune encephalomyelitis

**DOI:** 10.1084/jem.20190377

**Published:** 2019-08-29

**Authors:** Sheng Li, Yuqing Wu, Dongxue Yang, Chunyan Wu, Chunmei Ma, Xue Liu, Paul N. Moynagh, Bingwei Wang, Gang Hu, Shuo Yang

**Affiliations:** 1 Department of Immunology, Key Laboratory of Immunological Environment and Disease, State Key Laboratory of Reproductive Medicine, Nanjing Medical University, Nanjing, China; 2 Maynooth University Human Health Research Institute, Department of Biology, National University of Ireland Maynooth, Maynooth, Ireland; 3 Wellcome-Wolfson Institute for Experimental Medicine, Queen's University Belfast, Belfast, UK; 4 Department of Pharmacology, Nanjing University of Chinese Medicine, Nanjing, China

## Abstract

The NLRP3 inflammasome is critical for EAE pathogenesis; however, the role of gasdermin D (GSDMD), a newly identified pyroptosis executioner downstream of NLRP3 inflammasome, in EAE has not been well defined. Here, we observed that the levels of GSDMD protein were greatly enhanced in the CNS of EAE mice, especially near the areas surrounding blood vessels. GSDMD was required for the pathogenesis of EAE, and GSDMD deficiency in peripheral myeloid cells impaired the infiltration of immune cells into the CNS, leading to the suppression of neuroinflammation and demyelination. Furthermore, the loss of GSDMD reduced the activation and differentiation of T cell in the secondary lymphoid organs and prevented T cell infiltration into CNS of EAE. The administration of inflammasome-related cytokines partially rescued the impairment of pathogenesis of EAE in GSDMD KO mice. Collectively, these findings provide the first demonstration of GSDMD in peripheral myeloid cells driving neuroinflammation during EAE pathogenesis.

## Abstract (precis)

In this study, Li et al. demonstrate that gasdermin D in peripheral myeloid cells promotes the activation and differentiation of T cell in the secondary lymphoid organs, thus driving T cell–mediated neuroinflammation and demyelination in the CNS of EAE mice.

## Graphical Abstract

**Figure:**
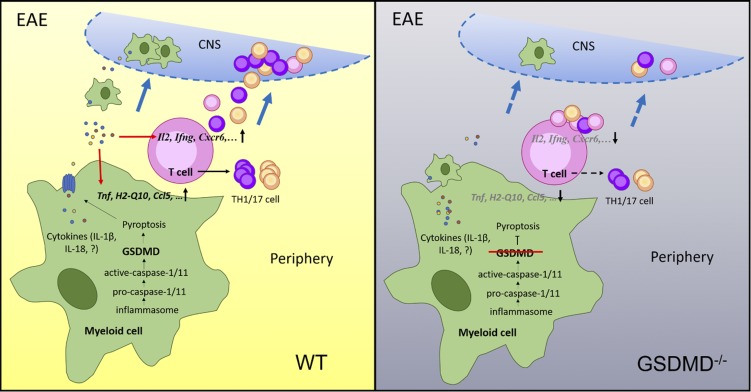

## Introduction

Multiple sclerosis (MS) is an incurable and progressive inflammatory disease of the central nervous system (CNS) with neuropathological features, including inflammatory demyelination, chronic axonal damage, and neurodegeneration (Frohman et al., 2006). However, the etiology of MS is not well understood, with the underlying basis to pathogenesis being ill-defined.

Experimental autoimmune encephalomyelitis (EAE) is the most commonly used animal model for studying MS since it shares neuropathological features with MS and can help to define the contribution of immune events to the development of MS (Ransohoff, 2012). EAE is mediated by myelin-specific T cells, which are activated in the peripheral lymphoid organs and then migrate into CNS by crossing the blood–brain barrier to mediate inflammatory responses, resulting in demyelination and neurodegeneration (Stromnes et al., 2008). Moreover, much evidence suggests that innate immune cells play critical roles during the process of EAE. Myeloid cells, such as dendritic cells (DCs) and macrophages, serve as APCs that mediate the cellular immune response by processing and presenting myelin antigens for recognition by T cells (Heppner et al., 2005; Chastain et al., 2011). Additionally, myeloid cells produce cytokines and chemokines that respectively facilitate the activation and differentiation of naive CD4^+^ T cells toward T helper type 1 (Th1) and Th17 cell lineages, and the translocation of effector T cells into CNS, all of which contributes to the progression of EAE. Furthermore, the peripheral myeloid cells themselves can be recruited into CNS and together with CNS-resident innate immune cells, such as microglia and astrocyte, facilitate neuroinflammation to cause neuron damage in EAE (Greter et al., 2005; Goverman, 2009; Chastain et al., 2011; Duffy et al., 2014).

Inflammasomes are cytosolic multiprotein complexes that are vital players of innate immunity and especially in the initiation of inflammatory responses in myeloid cells (Martinon et al., 2002). Upon recognition of stimuli, inflammasome-associated sensor proteins, together with apoptosis-associated speck-like protein containing CARD (ASC) and pro-inflammatory cysteinyl aspartate–specific proteinase (caspases; caspase-1 and -11), assemble into an oligomeric complex leading to caspase autoactivation, which controls the cleavage of pro–IL-1β and pro–IL-18 precursors into their mature forms and also induces a type of proinflammatory programmed cell death termed pyroptosis (Rathinam et al., 2012; Latz et al., 2013; Jorgensen and Miao, 2015). Notably, several independent studies showed that the expression of caspase-1, IL-1β, and IL-18 is elevated in the peripheral blood mononuclear cells and serum of MS patients (Huang et al., 2004). Furthermore, increasing recent evidence indicate that the NOD-like receptor protein 3 (NLRP3) inflammasome in APCs plays a critical role in the pathogenesis of EAE model by mediating chemotactic immune cell recruitment to the CNS (Gris et al., 2010; Inoue et al., 2012). Additionally, a recent study reported that Th17 cell–intrinsic ASC can promote the process of EAE by inducing IL-1β production via ASC–NLRP3–caspase-8 inflammasome axis (Martin et al., 2016). These studies highlight an important role for inflammasome in MS and EAE pathogenicity.

Gasdermin D (GSDMD) is a recently identified pore-forming protein, which mediates pyroptosis (Kayagaki et al., 2015; Shi et al., 2015). Proinflammatory caspases (caspase-1 and -4/5 in humans and caspase-1 and -11 in mice) can cleave GSDMD at its central linker domain, thereby releasing the autoinhibition by C-terminal GSDMD domain on the N-terminal GSDMD domain, causing N-terminal GSDMD domain fragments to translocate to the plasma membrane, where they oligomerize to form membrane pores, leading to lytic cell death or the secretion of processed IL-1β and IL-18 in living cells through pores (Ding et al., 2016; Liu et al., 2016; Evavold et al., 2018). Recent studies demonstrate that GSDMD plays a critical role in septic shock in response to bacterial infection, and in inflammasome-driven autoinflammatory diseases such as familial Mediterranean fever and neonatal-onset multisystem inflammatory disease (Kanneganti et al., 2018; Xiao et al., 2018). Interestingly, a recent study reported that GSDMD accumulation was observed in microglia and oligodendrocytes of CNS in both MS and EAE (McKenzie et al., 2018). However, to date there have been no reports on a pathophysiological role for GSDMD in neuroinflammation or EAE pathogenesis.

We now provide the first insight into the physiological function of GSDMD in EAE pathogenesis and describe the regulatory mechanism of GSDMD-mediated pyroptosis in the development of neuroinflammation during EAE. We discover that myeloid cell–intrinsic GSDMD is crucial for EAE pathogenesis by mediating the activation and differentiation of T cells in the secondary lymphoid organs, which facilitates T cell–mediated neuroinflammation and demyelination in the CNS.

## Results

### GSDMD-mediated pyroptosis is activated in the CNS of EAE

**Figure 1.**
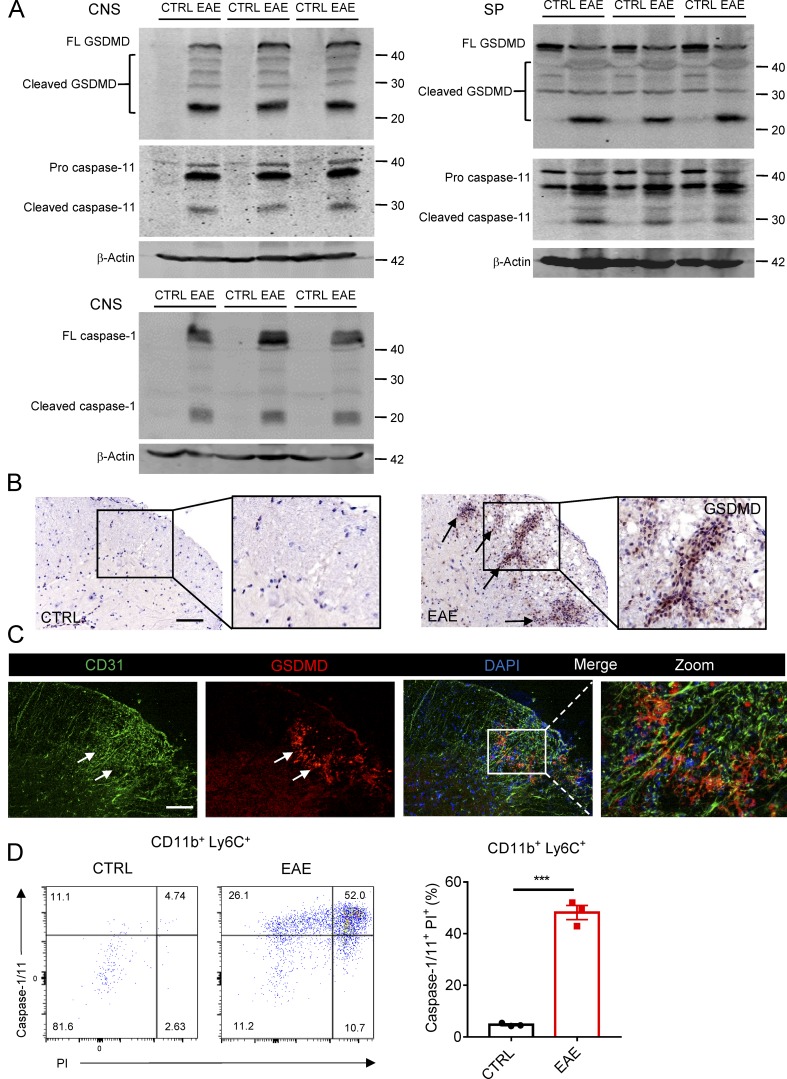
**GSDMD is strongly expressed and processed in CNS during EAE. (A)** Immunoblot analysis of full-length (FL) and cleaved GSDMD, caspase-11 and caspase-1 in the spinal cords and spleen (SP) from three pairs of control (CTRL) or EAE-induced WT mice at day 18 after immunization. **(B)** Immunohistochemistry images showing GSDMD expression in the spinal cord of control or EAE-induced WT mice at day 18 after immunization. Scale bar, 100 µm. **(C)** Immunofluorescent labeling of CD31 (green), GSDMD (red), and DAPI (blue) demonstrates the angiogenesis and the distribution of GSDMD (indicated by arrows) in the lesion area of spinal cord of EAE-induced WT mice at day 18 after immunization. Scale bar, 100 µm. **(D)** Flow-cytometric analysis of caspase-1/11^+^PI^+^ cells from CD11b^+^ Ly6C^+^ cells infiltrated to the spinal cord and brain of CTRL and MOG_35-55_-immunized WT mice at day 18 after immunization (*n* = 3 mice per group). Data are presented as a representative plot (left) and quantified percentages (right). Data are representative of three independent experiments. ***, P < 0.001. Error bars show means ± SEM. Unpaired *t* test for D.

To investigate whether GSDMD contributes to the pathogenesis in MS disease, we first tested the level of its expression in CNS, spleen, and LNs of an EAE mice model, which was induced by administration of myelin oligodendrocyte glycoprotein (MOG_35–55_) peptide and pertussis toxin. Immunoblotting analysis demonstrated greatly increased expression and cleavage of GSDMD and caspase-1/11 in the CNS and peripheral lymphoid organs of EAE-induced mice at the peak stage relative to untreated mice (Fig. 1 A and Fig. S1 A), suggesting that the pyroptosis mediated by GSDMD is indeed involved in the disease. Immunohistochemical analysis further confirmed the high-level expression of GSDMD in EAE CNS, and it was especially concentrated in lesion areas of spinal cord that were also characterized by strong cell infiltration. Interestingly, the positive staining of GSDMD was barely detectable in the CNS of control mice (Fig. 1 B). In addition, most of the cells highly expressing GSDMD and caspase-11 were detected in close proximity to the expanded capillaries at the edge of lesions in the spinal cord, as evidenced by closely adjacent expression of the endothelial marker CD31 (Fig. 1 C and Fig. S1 B), suggesting that these GSDMD-positive cells may represent cells recruited from the periphery. Moreover, FACS revealed a marked increase of caspase-1/11^+^ propidium iodide (PI)^+^ cells gated on the CD11b^+^ Ly6C^+^ (infiltrated peripheral monocytes/macrophages) in the CNS of mice during EAE compared with control mice (Fig. 1 D). Collectively, these data suggest that GSDMD-mediated pyroptosis is associated with EAE development, and its activation in CNS may be derived from infiltrating peripheral cells.

### GSDMD is essential for EAE induction

**Figure 2.**
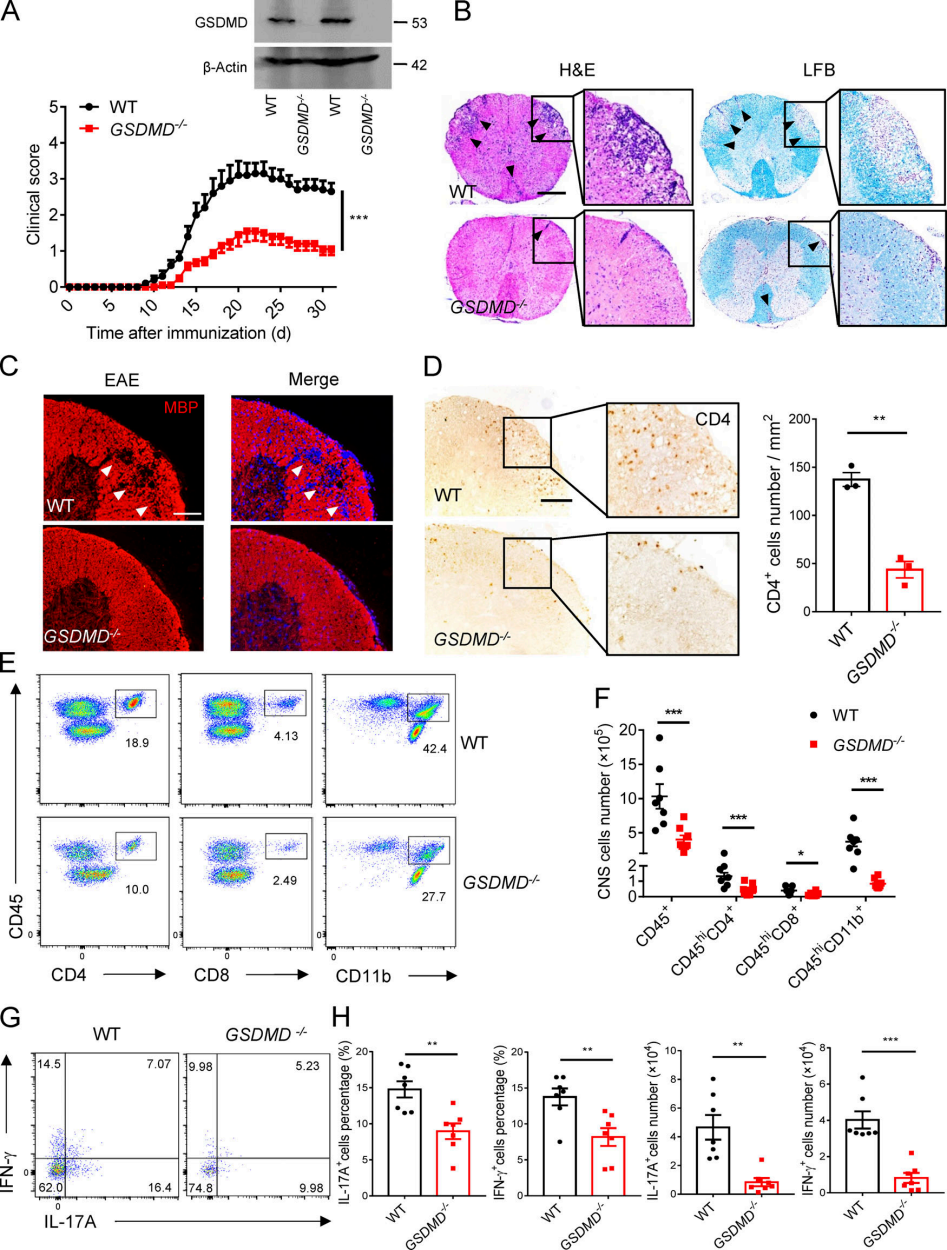
**GSDMD-deficient mice are resistant to EAE pathogenesis and neuroinflammation. (A)** Mean clinical scores of age-matched female WT and *GSDMD^−/−^* mice subjected to MOG_35-55_-induced EAE (*n* = 10 mice per group); immunoblot analysis of GSDMD expression in spleen from indicated mice. **(B)** H&E staining and LFB staining of spinal cord sections from EAE-induced WT and *GSDMD^−/−^* mice showing inflammatory cell infiltration and demyelination, respectively (arrowheads). Scale bar, 500 µm. **(C)** Immunofluorescent labeling of MBP (red) and DAPI (blue) visualizing the impairment of myelination (arrowheads) of EAE-induced WT and *GSDMD^−/−^* mice, respectively. Scale bar, 100 µm. **(D)** Immunohistochemistry analysis for infiltrating CD4^+^ T cells in the spinal cord of EAE-induced WT and *GSDMD^−/−^* mice (representative images on left and quantified cell numbers on right, *n* = 3 mice per group), respectively. Scale bar, 100 µm. **(E and F)** Flow-cytometric analysis of immune cells (including CD45^+^CD4^+^ T cells, CD45^+^CD8^+^ T cells, and CD45^+^CD11b^+^ monocytes) infiltrated to the spinal cord and brain of MOG_35-55_-immunized WT and *GSDMD^−/−^* mice at day 18 after immunization (*n* = 7 for WTs, *n* = 8 for *GSDMD^−/−^* mice). Data are presented as a representative plot (E) and summary graph of the absolute cell numbers (F). **(G and H)** Flow-cytometric analysis of Th1 (IFN-γ^+^) and Th17 (IL-17A^+^) cells from CD4^+^ T cells infiltrated to the spinal cord and brain of MOG_35-55_-immunized WT and *GSDMD^−/−^* mice at day 18 after immunization (*n* = 7 mice per group). Data are presented as a representative plot (G), quantified percentage, and absolute cell numbers (H). Data are pooled from three independent experiments. *, P < 0.05; **, P < 0.01; ***, P < 0.001. Error bars show means ± SEM. Unpaired *t* test for A, D, and H, and multiple unpaired *t* test for F.

To directly evaluate the requirement of GSDMD for EAE progression, we compared EAE in GSDMD KO mice and WT mice. We found that *GSDMD*^−/−^ mice were more resistant to EAE and developed less severe disease and clinical scores compared with WT mice (Fig. 2 A). Histopathological analysis (H&E staining and Luxol fast blue [LFB] staining) showed fewer infiltrating inflammatory cells and less demyelination in the spinal cord of *GSDMD*^−/−^ mice relative to their WT counterparts (Fig. 2 B). Myelin basic protein (MBP) is a protein that contributes to the process of myelination of nerves and maintenance of neural structure and is also as a marker of the myelin sheath of oligodendrocytes, with its loss being a major pathological feature of MS (Bradl and Lassmann, 2010). Immunofluorescence analysis showed regions of considerable diminution MBP expression at the peak of EAE disease in WT mice, but these were not apparent in *GSDMD*^−/−^ mice (Fig. 2 C). Moreover, *GSDMD*^−/−^ mice had significantly reduced numbers of activated macrophages, microglia, and astrocytes in EAE spinal cord compared with WT mice as determined by expression of the microglial and astrocyte cell markers, ionized calcium-binding adapter molecule 1 (IBA1), and glial fibrillary acidic protein (GFAP), respectively (Fig. S1, C and D). This indicates that GSDMD deficiency is associated with less severe neuroinflammation in EAE. Furthermore, fewer CD4^+^ T cells were detected at the edge of spinal cords of *GSDMD*^−/−^ mice relative to WT counterparts, further suggesting the impairment of recruitment of immune cells in *GSDMD*^−/−^ mice during EAE (Fig. 2 D). Consistent with immunostaining analysis, FACS revealed a marked reduction of T cells (CD45^+^, CD4^+^, and CD8^+^), myeloid cells, and activated microglia cells (CD45^+^, CD11b^+^) in the CNS of *GSDMD*^−/−^ mice during EAE compared with WT mice (Fig. 2, E and F). With respect to the population of CNS infiltrating CD4^+^ T cells, the percentages and absolute numbers of both Th1 and Th17 cells were greatly reduced in *GSDMD*^−/−^ mice (Fig. 2, G and H). Altogether, these data suggest that GSDMD is essential for EAE pathogenesis.

### GSDMD deficiency in peripheral myeloid cells suppresses neuroinflammation and pathogenesis of EAE

**Figure 3.**
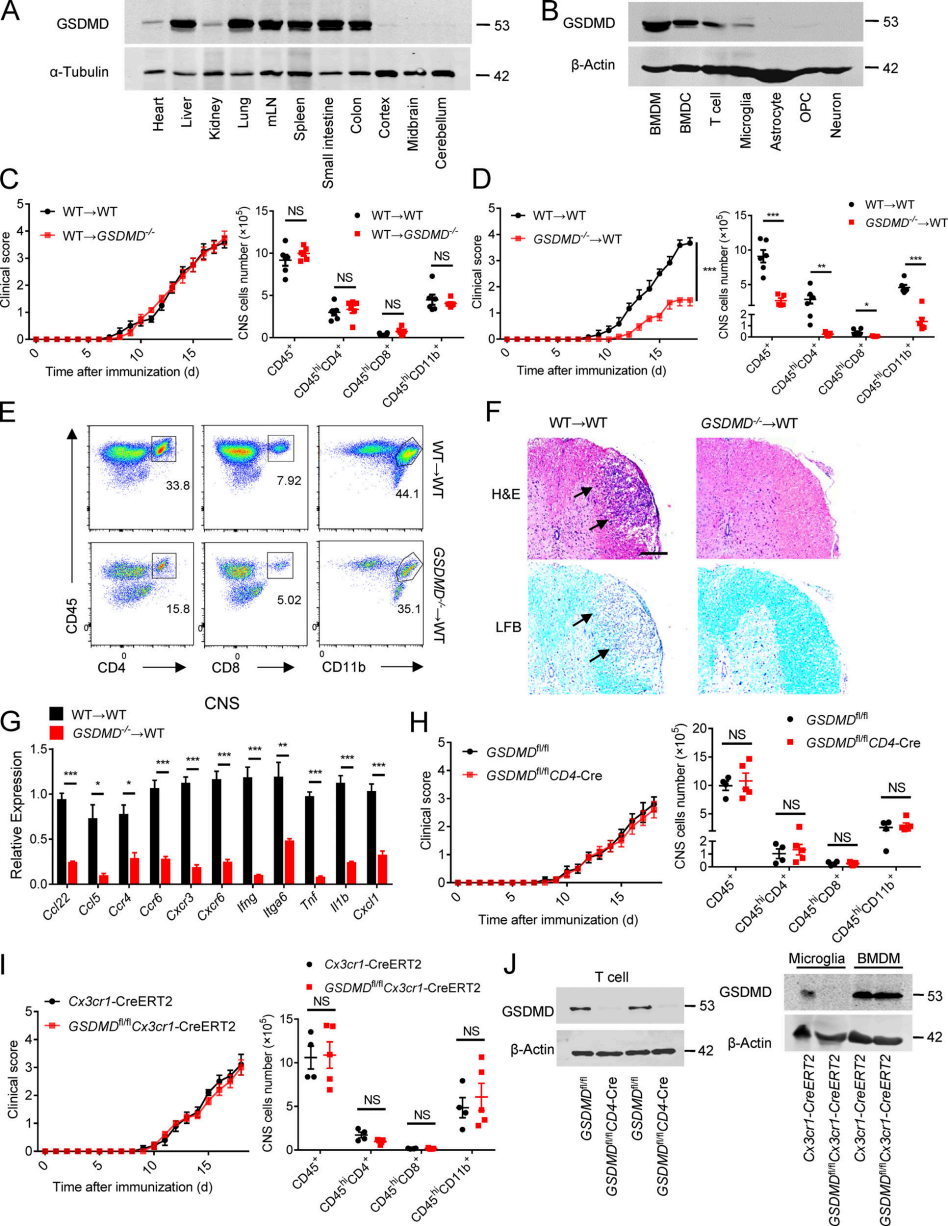
**GSDMD deletion in peripheral cells attenuates the development of EAE. (A)** Immunoblot analysis of the protein expression of GSDMD in different tissues (heart, liver, kidney, lung, mesenteric LNs [mLN], spleen, small intestine, colon, cortex, midbrain and cerebellum) from WT mice. **(B)** Immunoblot analysis of the protein expression of GSDMD in peripheral cells (BMDMs, bone marrow–derived DCs [BMDCs], and T cells) and CNS resident cells (microglia, astrocyte, oligodendrocyte progenitor cell [OPC], and neuron) from WT mice. **(C)** Mean clinical scores after EAE induction and summary graph of CNS-infiltrating immune cells at day 18 after immunization in WT and *GSDMD^−/−^* mice (*n* = 6 mice per group) adoptively transferred with WT bone marrow cells. **(D)** Mean clinical scores after EAE induction and summary graph of CNS-infiltrating immune cells at day 18 after immunization in WT mice (*n* = 5 or 6 mice per group) adoptively transferred with WT or *GSDMD^−/−^* bone marrow cells. **(E)** Representative plot from flow-cytometric analysis of immune cells infiltrated to the CNS (spinal cord and brain) of the mice in D. **(F)** H&E staining and LFB staining of spinal cord sections harvested from WT mice adoptively transferred with WT or *GSDMD^−/−^* bone marrow cells showing inflammatory cell infiltration and demyelination, respectively (arrows). Scale bar, 200 µm. **(G)** Quantitative PCR analysis of the relative mRNA expression of proinflammatory cytokines and chemokines in the spinal cord of WT mice (*n* = 4 mice per group) adoptively transferred with WT or *GSDMD^−/−^* bone marrow cells at the EAE peak. Data were normalized to a reference gene, Hprt. **(H)** Mean clinical scores after EAE induction and summary graph of CNS-infiltrating immune cells at day 18 after immunization in *GSDMD*^fl/fl^*CD4-*Cre and littermate control *GSDMD*^fl/fl^ mice (*n* = 4 or 5 mice per group). **(I)** Mean clinical scores after EAE induction and summary graph of CNS-infiltrating immune cells at day 18 after immunization in *GSDMD*^fl/fl^*Cx3cr1*-CreERT2 and *Cx3cr1*-CreERT2 mice (*n* = 4 or 5 mice per group). **(J)** Immunoblot analysis of GSDMD expression in T cells from indicated mice, and in microglia and BMDMs from indicated mice at 6 wk after administration of tamoxifen. Data are pooled from three independent experiments. *, P < 0.05; **, P < 0.01; ***, P < 0.001. Error bars show means ± SEM. Unpaired *t* test for C, D, H, and I, and multiple unpaired *t* test for C, D, and G–I.

To determine whether GSDMD deficiency in peripheral cells or CNS-resident cells contributes to the suppression of EAE, we measured the expression of GSDMD in various tissues, but it was strikingly low in CNS of homeostatic state (Fig. 3 A). Moreover, compared with peripheral cells (including bone marrow–derived macrophages [BMDMs], bone marrow–derived DCs, and T cells), microglia displayed very low expression of GSDMD with barely detectable expression other CNS cells including astrocytes, oligodendrocyte progenitor cells, and neurons (Fig. 3 B). Next, we generated bone marrow chimeric mice by adoptively transferring WT bone marrow cells into lethally irradiated WT or *GSDMD*^−/−^ recipient mice. Comparable EAE clinical scores were observed between these two recipients (Fig. 3 C), which also had comparable levels of infiltrated immune cells in the CNS (Fig. 3 C). However, a reverse bone marrow transfer experiment in which lethally irradiated WT recipient mice were reconstituted with bone marrow cells isolated from WT or *GSDMD*^−/−^ mice showed that WT mice reconstituted with the GSDMD deficiency bone marrow were more refractory to EAE induction than WT donors (Fig. 3 D). Additionally, the recruitment of peripheral immune cells into the CNS was reduced in *GSDMD*^−/−^ donors compared with WT donors (Fig. 3, D and E). H&E and LFB (Fig. 3 F) staining also confirmed that fewer infiltrating inflammatory cells and decreased demyelination were apparent in the spinal cord of *GSDMD*^−/−^ donor mice. Notably, the CNS of WT recipients reconstituted with the GSDMD^−/−^ bone marrow had significantly decreased expression of several inflammatory chemokines and cytokines known to mediate immune cells recruitment and neuroinflammation after the induction of EAE (Fig. 3 G). Thus, these results suggest that GSDMD functions in peripheral cells to promote EAE pathogenesis in the CNS.

We next examined if this role for GSDMD in peripheral cells was intrinsic to peripheral T cells by crossing the *GSDMD*^fl/fl^ mice with *CD4*-Cre mice to generate T cell conditional GSDMD KO mice. *GSDMD*^fl/fl^*CD4*-Cre and littermate control *GSDMD*^fl/fl^ mice developed similar clinical symptoms and immune cells infiltration after immunization (Fig. S2 and Fig. 3, H and J), suggesting that GSDMD in T cells has no role in driving EAE-associated pathogenesis. However, peripheral myeloid cells (DCs and macrophages) and CNS-resident microglia also contribute to neuroinflammation and pathogenesis in EAE (Duffy et al., 2014). To further explore the potential contribution of GSDMD in microglia to EAE pathogenesis, we crossed *GSDMD*^fl/fl^ mice with *Cx3cr1*-CreERT2 mice to generate microglia conditional GSDMD KO mice. Tamoxifen was administered to these mice to specifically delete GSDMD in microglia. This experimental design is based on a previous report showing that short-lived blood monocytes are renewed within 6 wk after tamoxifen administration, whereas GSDMD is specifically deleted in long-lived microglia of *GSDMD*^fl/fl^*Cx3cr1*-CreERT2 mice (Yona et al., 2013). GSDMD deficiency in microglia had no effect on the EAE phenotype (Fig. 3, I and J), and this is consistent with the bone marrow transfer experiments that concluded a lack of role for GSDMD in CNS resident cells. Together, these data demonstrate that deletion of GSDMD in peripheral myeloid cells might be responsible for the suppression of the pathogenesis and neuroinflammation of EAE.

### GSDMD deficiency impairs the priming and differentiation of T cell by myeloid cells and increases T cell accumulation in peripheral lymphoid organs during EAE

**Figure 4.**
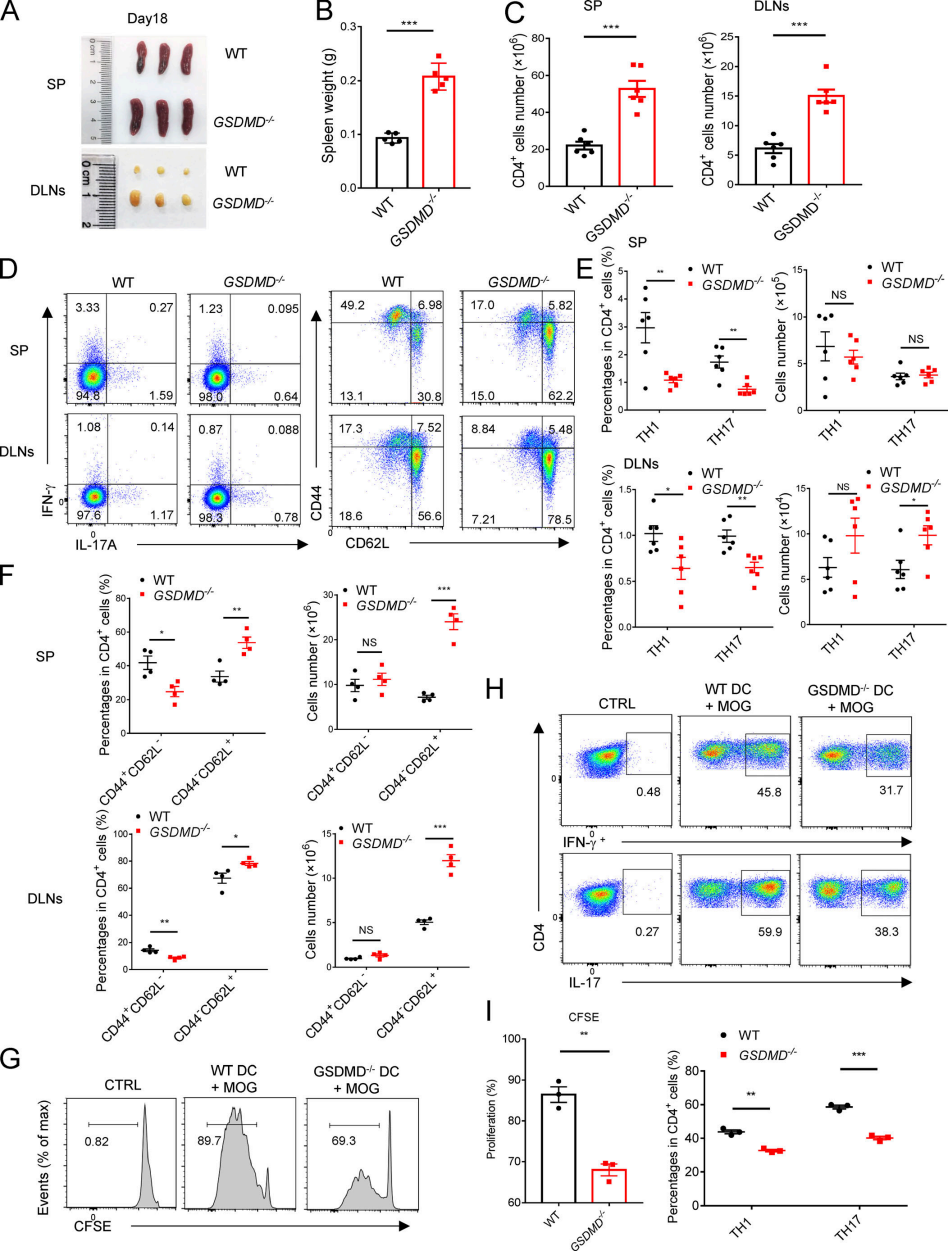
**The differentiation and egress of T cells in peripheral lymphoid organs during EAE are impaired in *GSDMD*^−/−^ mice. (A and B)** Spleens and DLNs harvested from three pairs of WTs and *GSDMD^−/−^* mice at day 18 after EAE induction (A) and quantified spleen (SP) weights (*n* = 5 mice per group; B). **(C)** Quantified absolute cell numbers of CD4^+^ T cells in spleen and DLNs from WTs and *GSDMD*^−/−^ mice at day 18 after EAE induction (*n* = 6 mice per group). **(D–F)** Flow-cytometric analysis of Th1 (IFN-γ^+^) and Th17 (IL-17A^+^) cells from CD4^+^ T cells in the spleen and DLNs of WT and *GSDMD^−/−^* mice (*n* = 6 mice per group) at day 18 after EAE induction, and effective T cell (CD44^+^CD62L^−^) and naive T cell (CD44^−^CD62L^+^) cells from CD4^+^ T cells in the spleen and DLNs of WT and *GSDMD^−/−^* mice (*n* = 4 mice per group) at day 18 after EAE induction. Data are presented as a representative plot (D), and quantified percentages and absolute cell numbers (E and F). **(G–I)** Flow-cytometric analysis of CFSE-labeled cells, Th1 (IFN-γ^+^) cells, and Th17 (IL-17A^+^) cells from CD4^+^ T cells at day 5 after in vitro cocultured with DCs (*n* = 3 per group). Data are presented as a representative plot (G and H) and quantified percentages (I). Data are pooled from three independent experiments (A–E) or from two independent experiments (F–I). *, P < 0.05; **, P < 0.01; ***, P < 0.001. Error bars show means ± SEM. Unpaired *t* test for B, C, and I, and multiple unpaired *t* test for E, F, and I. CTRL, control; max, maximum.

Antigen-activated T cells are key effector cells in the pathogenesis of EAE, which undergo activation in secondary lymphoid organs and then migrate into CNS due to the actions of cytokines and chemokines produced by APCs (Goverman, 2009). Given the phenotype observed above, we hypothesized that GSDMD in peripheral myeloid cells may be important for T cell response in peripheral lymphoid organs during EAE. Thus, decreased infiltration of CD4^+^ T cells in the CNS of *GSDMD*^−/−^ mice may be caused by decreased cellularity in the periphery. Unexpectedly, GSDMD-deficient mice show enlargement of spleen and draining LNs (DLNs) at the peak stage of EAE (Fig. 4, A and B), indicating the potential accumulation of T cells in peripheral lymphoid organs after immunization. Most notably, while the absolute numbers of CD4^+^ T cells (Fig. 4 C) were increased in the secondary lymphoid organs of *GSDMD*^−/−^ mice after EAE, the percentages of Th1 and Th17 cells were reduced, but their numbers were comparable in WT and *GSDMD*^−/−^ mice (Fig. 4, D and E). We next analyzed the naive CD62^+^CD44^−^CD4^+^ T cells (Th0) that have not been activated by APCs during EAE. Interestingly, the numbers of naive T cell in spleen and LNs were much higher in *GSDMD*^−/−^ mice after EAE than WT mice (Fig. 4, D and F), which might be responsible for the observed increase of CD4^+^ T cells in the secondary lymphoid organs of *GSDMD*^−/−^ mice. Given that we observed significantly reduced percentages of Th1 and Th17 cells in the secondary lymphoid organs of *GSDMD*^−/−^ mice during EAE, we suggest that GSDMD plays an important role in regulating T cell priming and differentiation in the peripheral lymphoid organs during EAE. This was directly addressed by performing in vitro T cell proliferation and differentiation assays that revealed *GSDMD*^−/−^ DCs to be greatly impaired in driving EAE MOG-induced T cell activation and differentiation relative to WT DCs (Fig. 4, G–I). Since the number of Th1 and Th17 cells in the secondary lymphoid organs was unaltered in *GSDMD*^−/−^ mice after EAE and the infiltration of Th1 and Th17 cells in the CNS was decreased (Fig. 2, G and H), we speculate that GSDMD deficiency also affects T cell migration into CNS. Additionally, we evaluated the profiles of T cells in thymus, spleen, and LN of *GSDMD*^−/−^ mice in homeostatic state. There were no significant differences observed between WT and *GSDMD*^−/−^ mice in the development of T cells and the profiles of naive T cell, effective T cell, and T regulatory (T reg) cell (Fig. S3). Therefore, these data suggest that GSDMD deficiency suppresses the priming and differentiation of T cells after the induction of EAE, meanwhile increasing T cell accumulation in peripheral lymphoid organs during disease.

**Figure 5.**
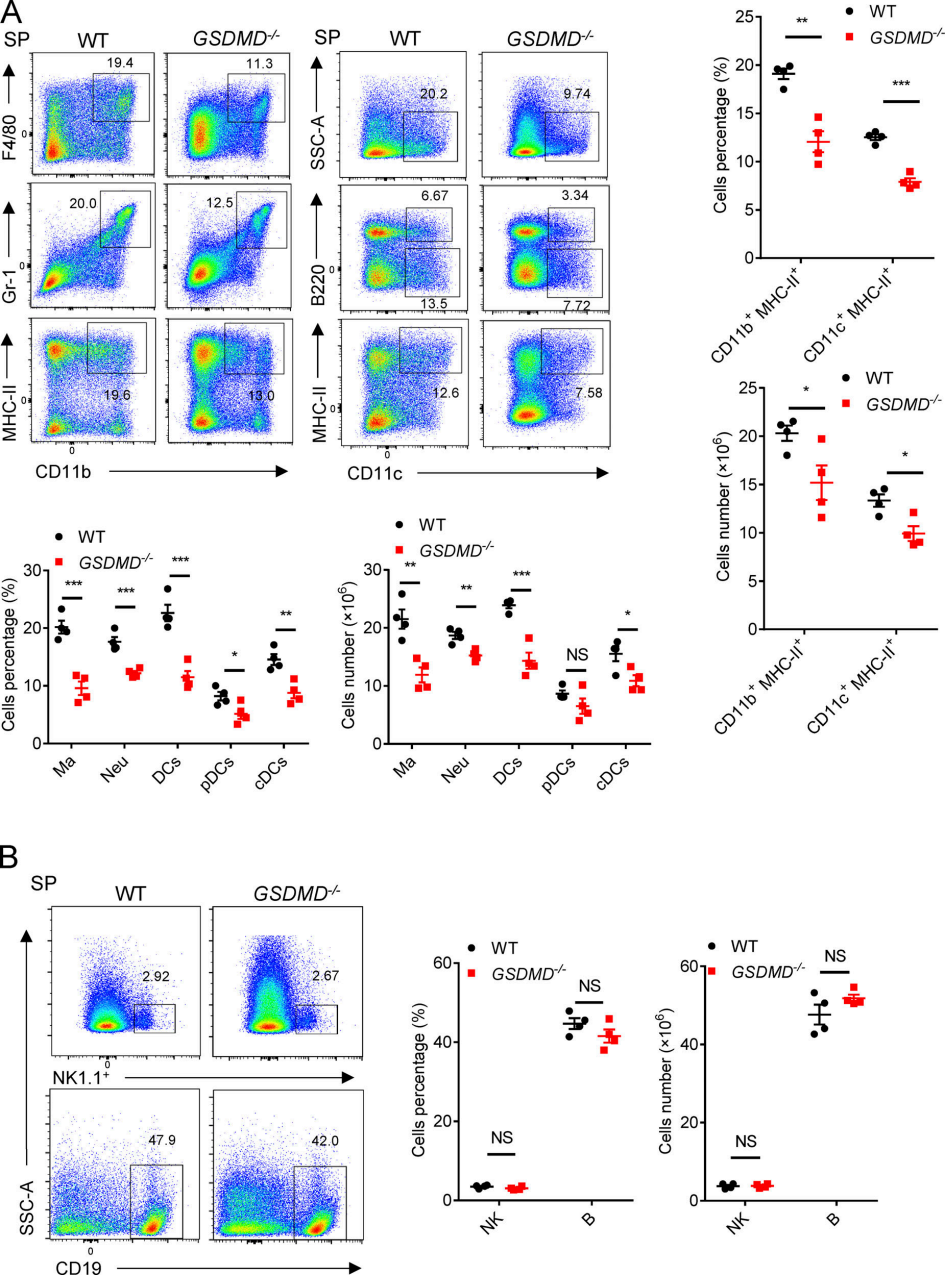
**Myeloid cells in peripheral lymphoid organs during EAE are impaired in *GSDMD*^−/−^ mice. (A)** Flow-cytometric analysis of macrophages (Ma; CD11b^+^F4/80^+^), neutrophils (Neu; CD11b^+^Gr-1^+^), DCs (CD11c^+^, including cDCs [CD11c^+^B220^−^] and pDCs [CD11c^+^B220^+^]), and MHCII-expressing cells (CD11b^+^MHCII^+^ and CD11c^+^MHCII^+^) in the spleen (SP) of WT and *GSDMD^−/−^* mice (*n* = 4 mice per group) at day 18 after EAE induction. Data are presented as a representative plot, quantified percentages, and absolute cell numbers. **(B)** Flow-cytometric analysis of NK cells (NK1.1^+^) and B cells (CD19^+^) in the spleen of WT and *GSDMD^−/−^* mice (*n* = 4 mice per group) at day 18 after EAE induction. Data are presented as a representative plot, quantified percentages, and absolute cell numbers. Data are pooled from two independent experiments. *, P < 0.05; **, P < 0.01; ***, P < 0.001. Error bars show means ± SEM. Multiple unpaired *t* test for A and B. SSC-A, side scatter–area.

To further address whether GSDMD functions on the myeloid cells to regulate T cell responses, we next investigated the composition of myeloid cells in the spleen during EAE. FACS revealed a marked reduction in the percentages and absolute numbers of macrophages (CD11b^+^F4/80^+^), neutrophils (CD11b^+^Gr-1^+^), and DCs (CD11c^+^, including classical DCs [cDCs; CD11c^+^B220^−^], and plasmacytoid DCs [pDCs; CD11c^+^B220^+^]) in the spleen of *GSDMD*^−/−^ mice during EAE relative to WT mice (Fig. 5 A). Importantly, the percentages and absolute numbers of MHCII-expressing cells (CD11b^+^MHCII^+^ and CD11c^+^MHCII^+^) in spleen were greatly reduced in EAE *GSDMD*^−/−^ mice (Fig. 5 A). In addition, GSDMD deficiency did not affect the number of B and natural killer (NK) cells in the spleen during EAE (Fig. 5 B). Moreover, we didn’t find any differences in the frequency and numbers of lymphocytic and myeloid cells in bone marrow, blood, and spleens of *GSDMD^−/−^* mice relative to WT controls under homeostatic conditions (Fig. S4). Thus, all these results further strengthen the conclusion of a key role for GSDMD in T cell priming by myeloid cells in the secondary lymphoid organs after EAE.

### GSDMD deficiency impairs the expression of genes required for T cell response in CD11b^+^ cells and CD4^+^ T cells of peripheral lymphoid organs during EAE

**Figure 6.**
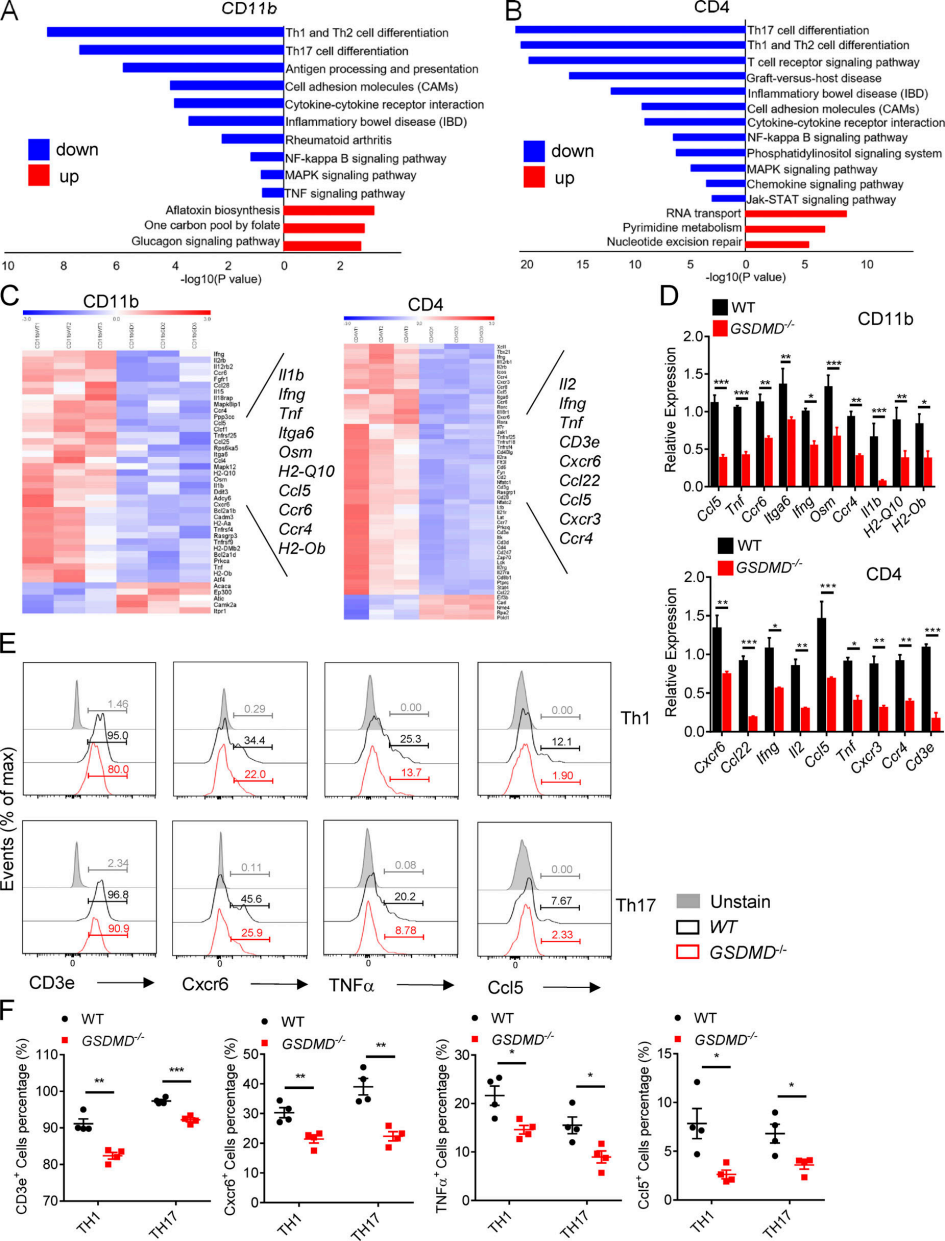
**GSDMD deficiency attenuates the expression of genes required for T cell response in CD11b^+^ cell and CD4^+^ T cell of peripheral lymphoid organs during EAE. (A and B)** KEGG analysis shows the most significantly enriched signaling pathways in CD11b^+^ macrophages (A) and CD4^+^ T cells (B) sorted from spleens of WT and *GSDMD^−/−^* mice at day 12 after EAE induction. **(C)** The heatmaps of genes with adjusted P value <0.05, false discovery rate <0.05, and log2 fold-change >1.2 from RNA-seq analysis of CD11b^+^ myeloid cells and CD4^+^ T cells isolated from spleens of three pairs of WTs and GSDMD^−/−^ mice at day 12 after EAE induction. **(D)** Quantitative PCR analysis of the indicated genes in CD11b^+^ myeloid cells and CD4^+^ T cells sorted from spleens in C (*n* = 3 mice per group). Data were normalized to a reference gene, Hprt. **(E and F)** Flow-cytometric analysis of CD3e^+^, Cxcr6^+^, TNFα^+^, and Ccl5^+^ cells from Th1 and Th17 cells in the spleen of WT and *GSDMD^−/−^* mice (*n* = 4 mice per group) at day 18 after EAE induction. Data are presented as a representative plot (E) and quantified percentages (F). Data are pooled from three independent experiments (E and F). *, P < 0.05; **, P < 0.01; ***, P < 0.001. Error bars show means ± SEM. Two-way ANOVA with Sidak’s multiple comparisons test for D. Multiple unpaired *t* test for F.

It is clear from the above results that peripheral myeloid cell–intrinsic GSDMD functions in peripheral lymphoid organs to regulate T cell response during EAE. To further dissect the mechanistic role of GSDMD in myeloid cells that regulates T cell responses, we performed RNA sequencing analysis using CD11b^+^ cells sorted from spleens of WT and *GSDMD*^−/−^ mice at the onset stage of EAE. Kyoto Encyclopedia of Genes and Genomes (KEGG) analysis showed the top pathways down-regulated in *GSDMD*^−/−^ CD11b^+^ cells mainly included Th cell differentiation, antigen processing and presentation, cell adhesion molecules, and cytokine signaling (Fig. 6 A). Consistently, the heatmap and real-time PCR analysis displayed significantly decreased expression in several genes associated with T cell response and migration, such as *Il1b*, *Ifng*, *Tnf*, *Osm*, *H2-Q10*, *H2-Ob*, *Itga6*, and *Ccl5* in *GSDMD*^−/−^ cells (Fig. 6, C and D). To further study the impact of GSDMD deficiency on T cell responses and determine whether T cells in *GSDMD*^−/−^ mice display an alteration in gene expression during EAE, we also performed RNA sequencing (RNA-seq) and examined the related genes expression in CD4^+^ T cells sorted from spleen. The top down-regulated pathways in *GSDMD*^−/−^ T cells after EAE induction were involved with Th cell differentiation, T cell receptor signaling pathway, and cytokine–chemokine signaling according to KEGG analysis (Fig. 6 B). Several genes associated with T cell activation and migration, such as *Il2, Ifng, Tnf, Cd3e, Ccl5, Ccl22, Ccr4, Cxcr3*, and *Cxcr6*, were down-regulated in *GSDMD*^−/−^ T cells at the onset of EAE as indicated in the heatmap and real-time PCR analysis (Fig. 6, C and D). Moreover, FACS revealed GSDMD deficiency greatly impaired the expression of Cxcr6, Ccl5, TNF-α, and CD3e expression on Th1 and Th17 cells during EAE (Fig. 6, E and F). Thus, these data further demonstrate that the expression of genes involved in T cell activation, differentiation, and migration in CD11b^+^ and CD4^+^ cells of peripheral lymphoid organs during EAE is significantly impaired by the deficiency of GSDMD.

### GSDMD in myeloid cells is required for the activation of CD4^+^ T cells during EAE

**Figure 7.**
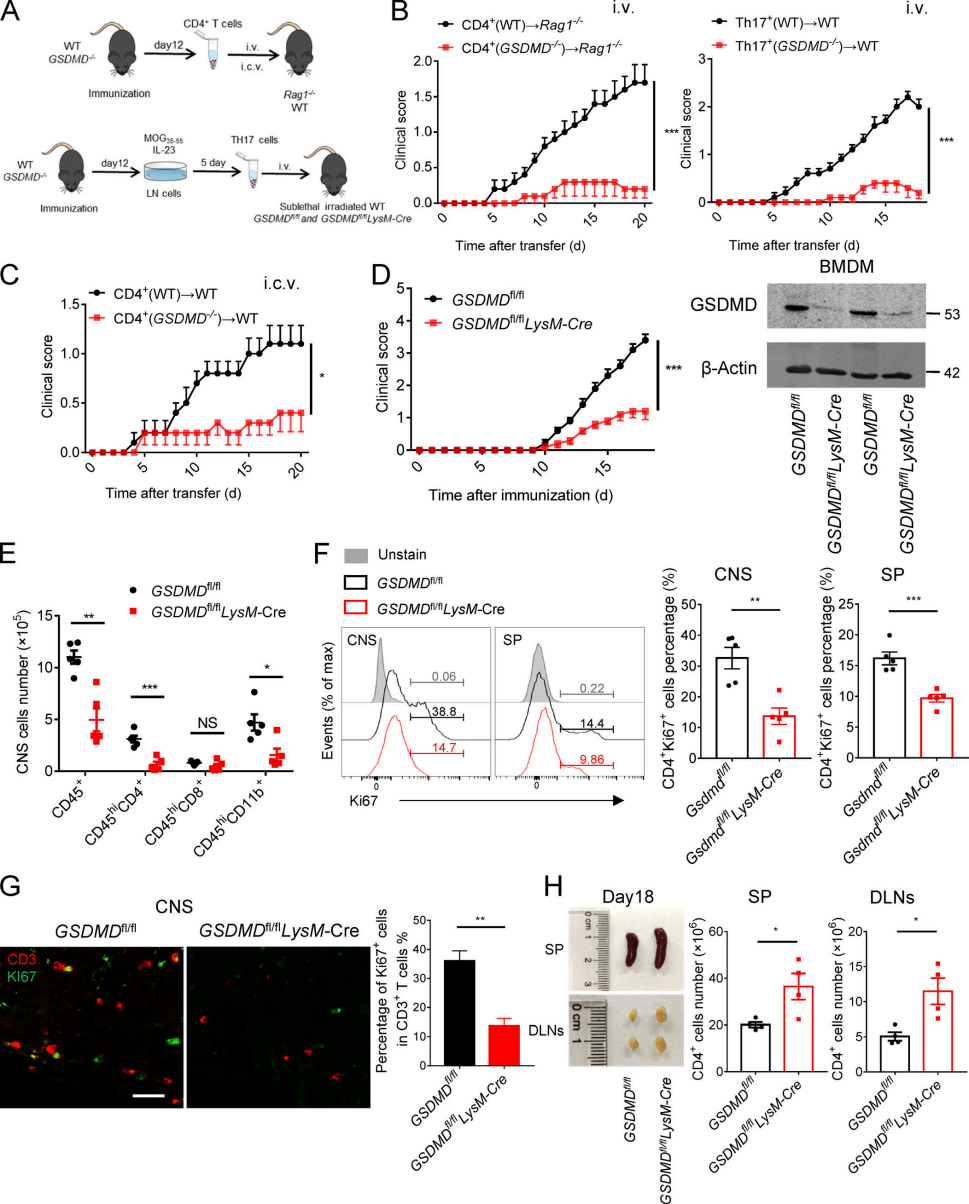
**GSDMD in peripheral myeloid cells is essential for the activation of CD4^+^ T Cells during EAE. (A)** Schematic representation of the experiments in B, C, and Fig. 8 G. CD4^+^ T cells were obtained from spleens of WT or *GSDMD^−/−^* mice at day 12 after EAE induction, then transferred into *Rag1*^−/−^ mice by i.v. injection or into WT mice by i.c.v. injection. DLN cells were obtained from WT or *GSDMD^−/−^* mice at day 12 after EAE induction, then cultured in vitro with MOG_35-55_ and IL-23 under Th17 cell–polarizing conditions before transfer into sublethal irradiated WTs or *GSDMD*^fl/fl^ and *GSDMD*^fl/fl^*LysM*-Cre mice, respectively. **(B and C)** Mean clinical scores after CD4 T cell and Th17 cell transfer by i.v. (B) and CD4 T cell transfer by i.c.v. (C; *n* = 5 mice per group). **(D)** Mean clinical scores after EAE induction in *GSDMD*^fl/fl^*LysM*-Cre and littermate control *GSDMD*^fl/fl^ mice (*n* = 5 mice per group); immunoblot analysis of GSDMD expression in BMDMs from indicated mice. **(E)** Summary graph of CNS-infiltrating immune cells at day 18 after immunization in *GSDMD*^fl/fl^*LysM*-Cre and littermate *GSDMD*^fl/fl^ mice (*n* = 5 mice per group). **(F)** Flow-cytometric analysis of KI67^+^ cells among CD4^+^ T cells in the CNS and spleens (SP) of *GSDMD*^fl/fl^*LysM*-Cre and littermate *GSDMD*^fl/fl^ mice at day 18 after immunization (*n* = 5 mice per group). **(G)** Immunofluorescent labeling of CD3 (red) and KI67 (green) demonstrate the proliferation of T cells in the spinal cord of indicated mice at day 18 after immunization. Data are presented as a representative image (left) and quantified percentage (right). Scale bar, 50 µm. **(H)** Quantified absolute cell numbers of CD4^+^ T cell in spleen and DLNs from *GSDMD*^fl/fl^*LysM*-Cre and littermate control *GSDMD*^fl/fl^ mice at day 18 after EAE induction (*n* = 4 mice per group). Data are pooled from three independent experiments (A–G) or from two independent experiments (H). *, P < 0.05; **, P < 0.01; ***, P < 0.001. Error bars show means ± SEM. Unpaired *t* test for B–D and F–H, and multiple unpaired *t* test for E.

To further test the impact of GSDMD deficiency on T cell function during EAE, CD4^+^ T or Th17 cells isolated from spleens of WT and *GSDMD*^−/−^ mice at the time of EAE onset (day 12) were i.v. transferred to *Rag1*^−/−^ or C57BL/6 WT recipients (Fig. 7 A). CD4^+^ T cells from immunized *GSDMD*^−/−^ mice failed to induce passive EAE in *Rag1*^−/−^ or WT recipients, but CD4^+^ T cells obtained from WT immunized mice promoted mild EAE (Fig. 7 B). This indicated that GSDMD deficiency results in functional impairment of T cells during EAE induction. Next, to further investigate whether GSDMD deficiency affects T cell migration, we directly transferred CD4^+^ T cells into the brain of WT mice by intracerebroventricular (i.c.v.) injection in order to bypass the migration of T cells from peripheral to CNS. Again, CD4^+^ T cells obtained from immunized *GSDMD*^−/−^ mice developed lower levels of EAE pathogenesis compared with immunized WT mice (Fig. 7 C), suggesting that the critical function of GSDMD may reside in regulating the activation and/or differentiation of T cells during EAE.

**Figure 8.**
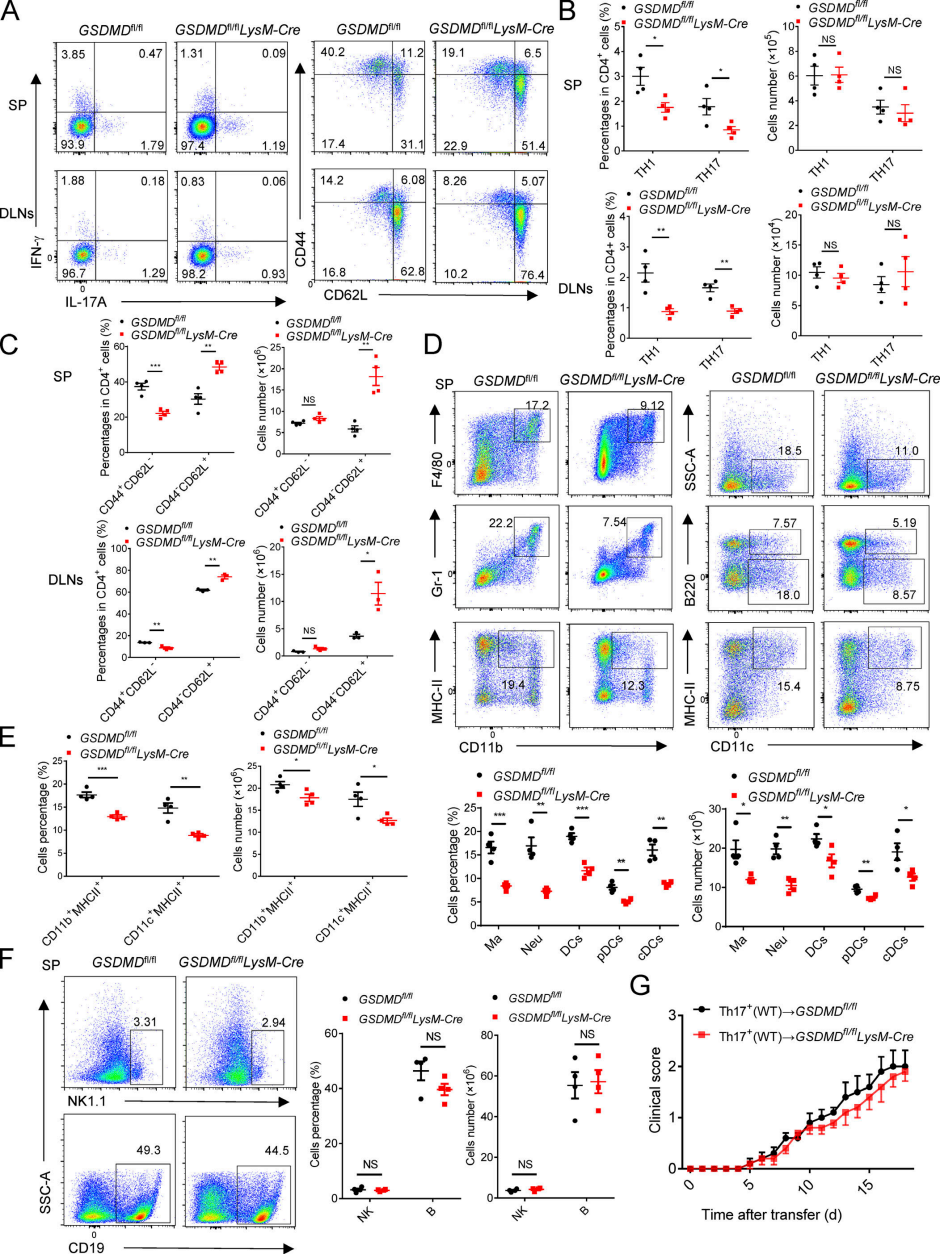
**GSDMD in peripheral myeloid cells is essential for T cell priming by myeloid cells during EAE. (A–C)** Flow-cytometric analysis of Th1 (IFN-γ^+^) and Th17 (IL-17A^+^) cells, effective T cells (CD44^+^CD62L^−^), and naive T cells (CD44^−^CD62L^+^) from CD4^+^ T cells in the spleen (SP) and DLNs of *GSDMD*^fl/fl^*LysM*-Cre and littermate control *GSDMD*^fl/fl^ mice (*n* = 4 mice per group) at day 18 after EAE induction. Data are presented as a representative plot (A), and quantified percentages and absolute cell numbers (B and C). **(D and E)** Flow-cytometric analysis of macrophages (Ma; CD11b^+^F4/80^+^), neutrophils (Neu; CD11b^+^Gr-1^+^), DCs (CD11c^+^, including cDCs [CD11c^+^B220^−^] and pDCs [CD11c^+^B220^+^]), and MHCII-expressing cells (CD11b^+^MHCII^+^ and CD11c^+^MHCII^+^) in the spleen of *GSDMD*^fl/fl^*LysM*-Cre and littermate control *GSDMD*^fl/fl^ mice (*n* = 4 mice per group) at day 18 after EAE induction. Data are presented as a representative plot (D), and quantified percentages and absolute cell numbers (D and E). **(F)** Flow-cytometric analysis of NK cells (NK1.1^+^) and B cells (CD19^+^) in the spleen of *GSDMD*^fl/fl^ and *GSDMD*^fl/fl^*LysM-*Cre mice (*n* = 4 mice per group) at day 18 after EAE induction. Data are presented as a representative plot, quantified percentages and absolute cell numbers. **(G)** Mean clinical scores after Th17-cell transferring by i.v. to indicated mice (*n* = 5 mice per group). Data are pooled from two independent experiments. *, P < 0.05; **, P < 0.01; ***, P < 0.001. Error bars show means ± SEM. Unpaired *t* test for G and multiple unpaired *t* test for B, C, E, and F. SSC-A, side scatter–area.

Given that we have earlier shown that GSDMD in peripheral myeloid cells is responsible for the neuroinflammation and pathogenesis of EAE, we next examined if peripheral myeloid cell–intrinsic GSDMD plays a function in T cell activation during EAE. To this end, we generated myeloid cell conditional GSDMD-KO mice by crossing the *GSDMD*^fl/fl^ mice with *LysM*-Cre mice. Since these conditional KO mice lack GSDMD expression in myeloid cells and we have already excluded the effect of GSDMD in microglia using *Cx3cr1*-CreERT2 mice (Fig. 3, I and J), the *LysM*-Cre mice allowed for direct investigation of the role of GSDMD in peripheral myeloid cells in T cell activation after EAE induction. Notably, the EAE clinical scores and infiltration of immune cells into CNS were considerably diminished in *GSDMD*^fl/fl^*LysM*-Cre mice relative to *GSDMD*^fl/fl^ mice (Fig. 7, D and E). Moreover, T cell proliferation was significantly impaired in the CNS and spleens of *GSDMD*^fl/fl^*LysM*-Cre mice, as shown by reduced staining of the proliferation marker CD4^+^Ki67^+^ at the EAE peak stage (Fig. 7, F and G). Additionally, we observed a significant increase in the number of CD4 T cells in the spleen and DLNs of *GSDMD*^f/f^ *LysM*-Cre mice during EAE relative to littermate controls (Fig. 7 H), but a marked reduction in the percentages of Th1 and Th17 cells (Fig. 8, A and B). Moreover, the number of naive CD62^+^CD44^−^CD4^+^(Th0) T cells in spleen and LNs was much higher in *GSDMD*^f/f^ *LysM*-Cre mice after EAE than tissues from controls (Fig. 8, A and C). In addition, the percentages and numbers of macrophages, neutrophils, DCs, and MHCII-expressing cells were greatly reduced in the spleen of *GSDMD*^f/f^ *LysM*-Cre mice during EAE (Fig. 8, D and E). We didn’t find any differences in the frequency and numbers of lymphocytic and myeloid cells in bone marrow, blood, and spleens of *GSDMD*^f/f^ *LysM*-Cre mice relative to littermate controls (Fig. S5). Furthermore, GSDMD deficiency in myeloid cells did not affect the number of B and NK cells in the spleen during EAE (Fig. 8 F). Thus, all of these data strongly suggest that GSDMD in peripheral myeloid cells is essential for T cell activation and ensuing pathogenesis in EAE. To further exclude an effect of GSDMD deficiency in microglia, we next performed adoptive transfer studies in *GSDMD*^f/f^ *LysM*-Cre mice and their littermate controls by adoptive transfer of Th17 cells from immunized WT mice. We found that MOG-reactive Th17 cells induced comparable EAE clinical scores between *GSDMD*^f/f^ *LysM*-Cre mice and controls (Fig. 8 G), which further suggests that GSDMD in peripheral myeloid cells but not in CNS resident microglia is essential for pathogenesis in EAE.

### Pharmacological inhibition of GSDMD attenuates EAE, and inflammasome-related cytokines promote EAE pathogenesis in GSDMD-deficient mice

**Figure 9.**
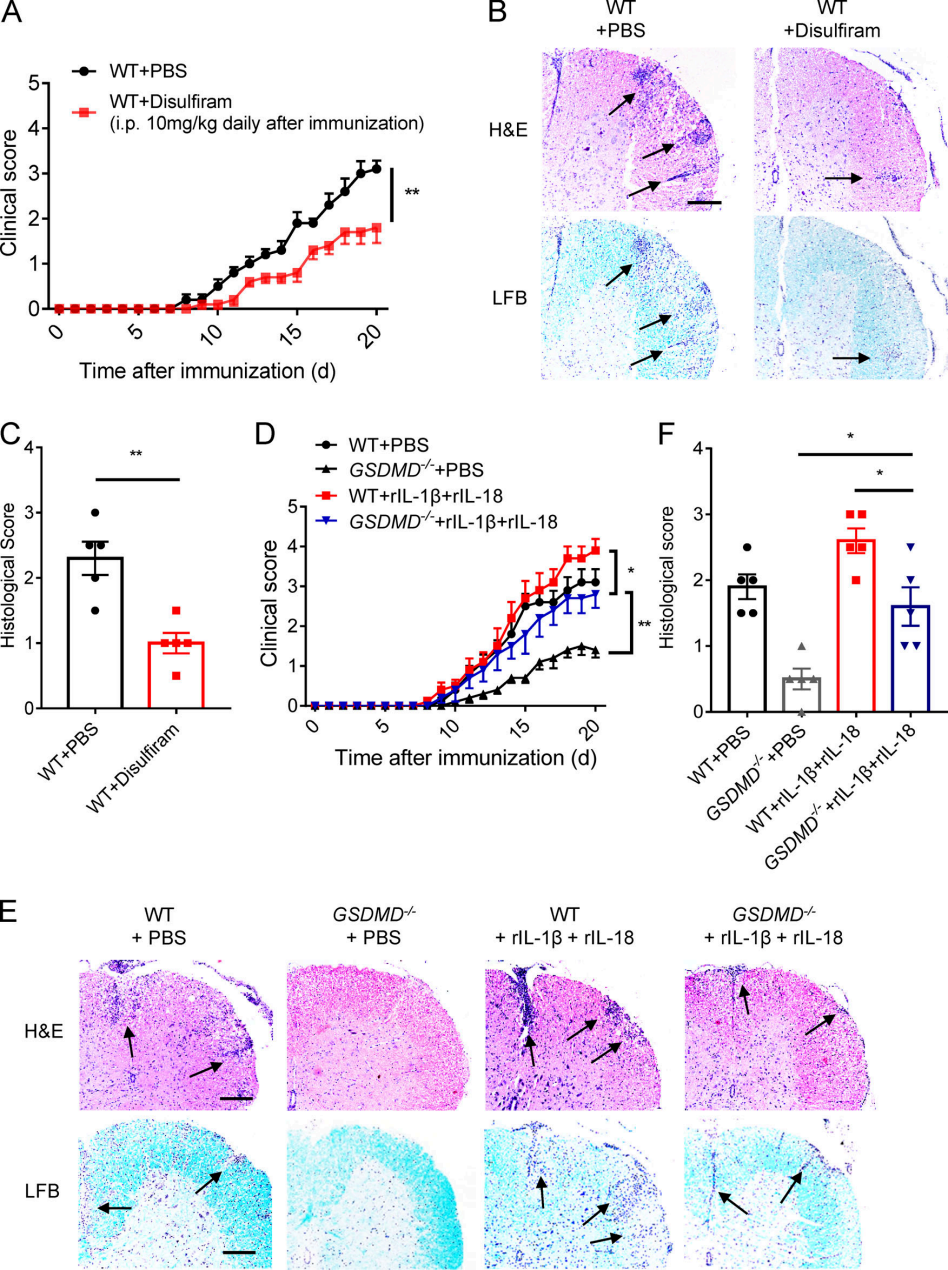
**Pharmacological inhibition of GSDMD attenuates EAE, and inflammasome-related cytokines promote EAE pathogenesis in GSDMD-deficient mice. (A)** Mean clinical scores of WT mice injected (i.p.) with disulfiram or PBS daily after EAE induction, respectively (*n* = 5 mice per group). **(B and C)** Representative images (B) and histological scores (C) of H&E staining and LFB staining of spinal cord sections from mice in A. Inflammatory cell infiltration and demyelination indicated by arrow. Scale bar, 200 µm. **(D)** Mean clinical scores of WTs and *GSDMD^−/−^* mice which were i.v. injected with rIL-1β and rIL-18 combination or PBS at day 4, 8, 12, and 16 after EAE induction, respectively (*n* = 5 mice per group). **(E and F)** Representative images (E) and histological scores (F) of H&E staining and LFB staining of spinal cord sections from mice in D. Inflammatory cell infiltration and demyelination indicated by arrow. Scale bars, 200 µm. Histological scores in C and F: 0, no inflammatory cell infiltration and no demyelination; 1, slight inflammatory cell infiltration or demyelination observed; 2, moderate inflammatory cell infiltration or demyelination in several spots; 3, substantial inflammatory cell infiltration and large area of demyelination. Data are pooled from three independent experiments (D–F) or from two independent experiments (A–C). *, P < 0.05; **, P < 0.01. Error bars show means ± SEM. Unpaired *t* test for A, C, D, and F.

Given that GSDMD-deficient mice show impaired pathogenesis, we were keen to assess if pharmacological inhibition of GSDMD could phenocopy the genetic models. We thus directly assessed the effects of pyroptosis inhibition on EAE progression in vivo by i.p. administration with disulfiram, a recently described potent inhibitor of GSDMD (Hu et al., 2018). We found that disulfiram treatment protected against EAE development and greatly attenuated the clinical and histopathological scores (Fig. 9, A–C). Thus, this result further indicates an important role of GSDMD in the pathogenesis of EAE and suggests a potential therapeutic use of targeting GSDMD-mediated pyroptosis in MS treatment. Different reports have shown that IL-1β and IL-18, the downstream cytokines of inflammasome activation, have high-level expression in MS patients and promote T cell differentiation, survival, and migration during EAE (Lalor et al., 2011; Inoue et al., 2012; Martin et al., 2016). Moreover, GSDMD can facilitate the secretion of IL-1β and IL-18 through cell lysis by pyroptosis or forming pores in living cells (Shi et al., 2015; Evavold et al., 2018). To understand whether the impairment of EAE pathogenesis in GSDMD-deficient mice is completely dependent on the loss of inflammasome-related cytokines, we assessed the effects of IL-1β and IL-18 on EAE progression in vivo by i.v. administration of their recombinant proteins in WT and *GSDMD*^−/−^ mice immunized with MOG. Although administration of recombinant IL-1β (rIL-1β) and rIL-18 in *GSDMD*^−/−^ mice developed more severe EAE as demonstrated by higher clinical and histopathological scores compared with their PBS-control mice, the disease severity of *GSDMD*^−/−^ mice still was milder than WTs even after administration with rIL-1β and rIL-18 (Fig. 9, D–F). It is hardly surprising that this exogenous supplementation exacerbates pathology in WTs since the exogenous levels of these cytokines likely greatly exceed the endogenous levels under conditions of EAE, and maximal pathogenesis is limited by the endogenous levels of IL-1β and IL-18. Importantly, the exacerbation of EAE severity by these cytokines in GSDMD KO mice did not completely reach the severity observed in WT PBS control mice. Thus, these results indicate that administration of IL-1β and IL-18 can only partially rescue the impairment of EAE pathogenesis in *GSDMD*^−/−^ mice, which suggests that the pathogenic role of GSDMD may also be dependent on the release of other factors in response to the pyroptotic actions of GSDMD.

## Discussion

GSDMD protein, a newly identified pyroptosis executioner downstream of inflammasome activation, has been reported to accumulate in the CNS of MS (McKenzie et al., 2018), but it is not clear what the physiological role and mechanism of GSDMD is in the EAE pathogenesis. In this study, we found that the ablation of GSDMD in peripheral myeloid cells reduced the infiltration of immune cells into the CNS and conferred robust protection from EAE development in mice immunized with MOG_35-55_. In addition, GSDMD deficiency impaired the activation and differentiation of T cell in the secondary lymphoid organs, thus preventing T cell–mediated neuroinflammation in EAE.

While the recent study suggested GSDMD-mediated pyroptosis appeared in the CNS of patients with MS and in EAE mice (McKenzie et al., 2018), the issues of the relative contribution of peripheral versus CNS GSDMD to disease pathology were unknown. The conclusion that GSDMD-mediated pyroptosis involves microglia during EAE progression cannot be addressed only by GSDMD immunoreactivity in Iba-1 immuno-positive cells in that study because of Iba-1 expression in both peripheral and CNS macrophages (Müller et al., 2015). In fact, both peripheral and CNS macrophages contribute to EAE progression (Bauer et al., 1995; Xiao et al., 2013; Duffy et al., 2014). Here we found that GSDMD in peripheral cells is indispensable for EAE induction by using the bone marrow chimeric EAE models in which the bone marrow from *GSDMD*^−/−^ mice exhibits impaired capability to induce neuroinflammation and pathogenesis of EAE. Moreover, GSDMD-specific deletion in microglia by using the *GSDMD*^fl/fl^ *Cx3cr1*-CreERT2 mice does not suppress CNS autoimmune inflammation after EAE induction, suggesting that GSDMD in microglia does not play a major role in regulating EAE pathogenesis. Notably, we observed an abundance of GSDMD immunoreactivity near the areas surrounding blood vessels of spinal cords within EAE, indicating that peripheral cells might be recruited and undergo pyroptosis in the diseased brain. Additionally, the studies in *GSDMD*^f/f^ *LysM*-Cre mice also showed that GSDMD deficiency in myeloid cells attenuated the progression of EAE. Therefore, our studies provide the first demonstration of a peripheral myeloid–intrinsic role for GSDMD in driving neuroinflammation in EAE.

Our RNA-seq analysis revealed that the major immune pathways down-regulated in CD11b^+^ cells from immunized *GSDMD*^−/−^ mice were associated with T cell response, including Th1 and Th17 cell differentiation, antigen processing and presentation, cell adhesion molecules, and cytokine signaling, which indicated that GSDMD-sufficient APCs are essential for priming T cell immune response. The heatmap results summarized that GSDMD deficiency severely impaired the expression of several genes for T cell activation and differentiation, such as *Tnf*, *Ifng*, *Il1b*, *H2-Q10*, and *H2-Ob*. TNF-α, IFN-γ, and IL-1β are key pro-inflammatory cytokines for the expansion and differentiation of reactive T cells in the secondary lymphoid organs, and an abundance of previously published reports demonstrated their critical roles in EAE development (Hirota et al., 2011; Raphael et al., 2015). H2-Q10 and H2-Ob are certain MHC haplotypes, and the importance of specific MHC molecules in determining EAE susceptibility has been well established (Weissert et al., 1998; Irla et al., 2010). In addition, the significantly reduced expression of genes for chemotaxis and adhesion, including *Ccl5*, *Ccr4*, *Ccr6*, and *Itga6,* was observed in splenic CD11b^+^ cells from immunized *GSDMD*^−/−^ mice. There have been a number of reports that chemotaxis molecules that enhance immune cell migration are strongly connected with EAE progression (Inoue et al., 2012; Poppensieker et al., 2012; Cheng and Chen, 2014). For CD4^+^ T cell from EAE mice, KEGG pathway analysis showed that the biological terms down-regulated in GSDMD^−/−^ cells majorly include Th1 and Th17 cell differentiation, T cell signaling pathway, cell adhesion molecules, and cytokine–chemokine signaling. Significant reductions in mRNA levels of *Il2, Ifng, Tnf, Cd3e, Ccl5, Ccl22, Ccr4, Cxcr3*, and *Cxcr6* were identified in splenic CD4^+^ T cells from *GSDMD*^−/−^ mice. IL-2 and CD3e are necessary for the expansion and survive of reactive T cells (Tamura and Nariuchi, 1992; Liao et al., 2013). TNF-α and IFN-γ can drive and amplify T cell response and inflammation during EAE pathogenesis (Raphael et al., 2015). Ccl5, Ccl22, Ccr4, Cxcr3, and Cxcr6 are migration-related molecules, which are required for T cells to enhance their migration ability to the CNS during the development of EAE (dos Santos et al., 2005; Inoue et al., 2012). Therefore, GSDMD-mediated pyroptosis up-regulates the expression of genes required for T cell response in the secondary lymphoid organs, which are involved in development of EAE and probably in MS as well.

Although RNA-seq results indicated that GSDMD deficiency might affect T cell migration into CNS, direct injection of CD4^+^ T cell from immunized *GSDMD*^−/−^ mice injection into the CNS induced much milder EAE compared with that induced by WT CD4^+^ T cells, suggesting that T cell activation is likely to be the substantive pathway affected by GSDMD deficiency during EAE. Consistent with this, our studies in *GSDMD*^f/f^ *LysM*-Cre mice further showed that the protective effect of GSDMD deficiency in myeloid cells was associated with the attenuation of T cell activation and proliferation during EAE. We demonstrate that the administration of IL-1β and IL-18 only partially rescued the impairment of EAE pathogenesis in *GSDMD*^−/−^ mice. Notably, a recent study showed that GSDMD deficiency promotes cyclic GMP-AMP synthase–dependent IFN production by reducing intracellular potassium (K^+^) efflux through GSDMD-formed pores (Banerjee et al., 2018). IFN-β administration is a first-line disease-modifying therapy for MS and thought to control CNS inflammation and neurodegeneration (Banerjee et al., 2018). Thus, whether the effects of GSDMD on IFN production are involved in GSDMD-mediated EAE progression needs to be further investigated in the future.

**Figure 10.**
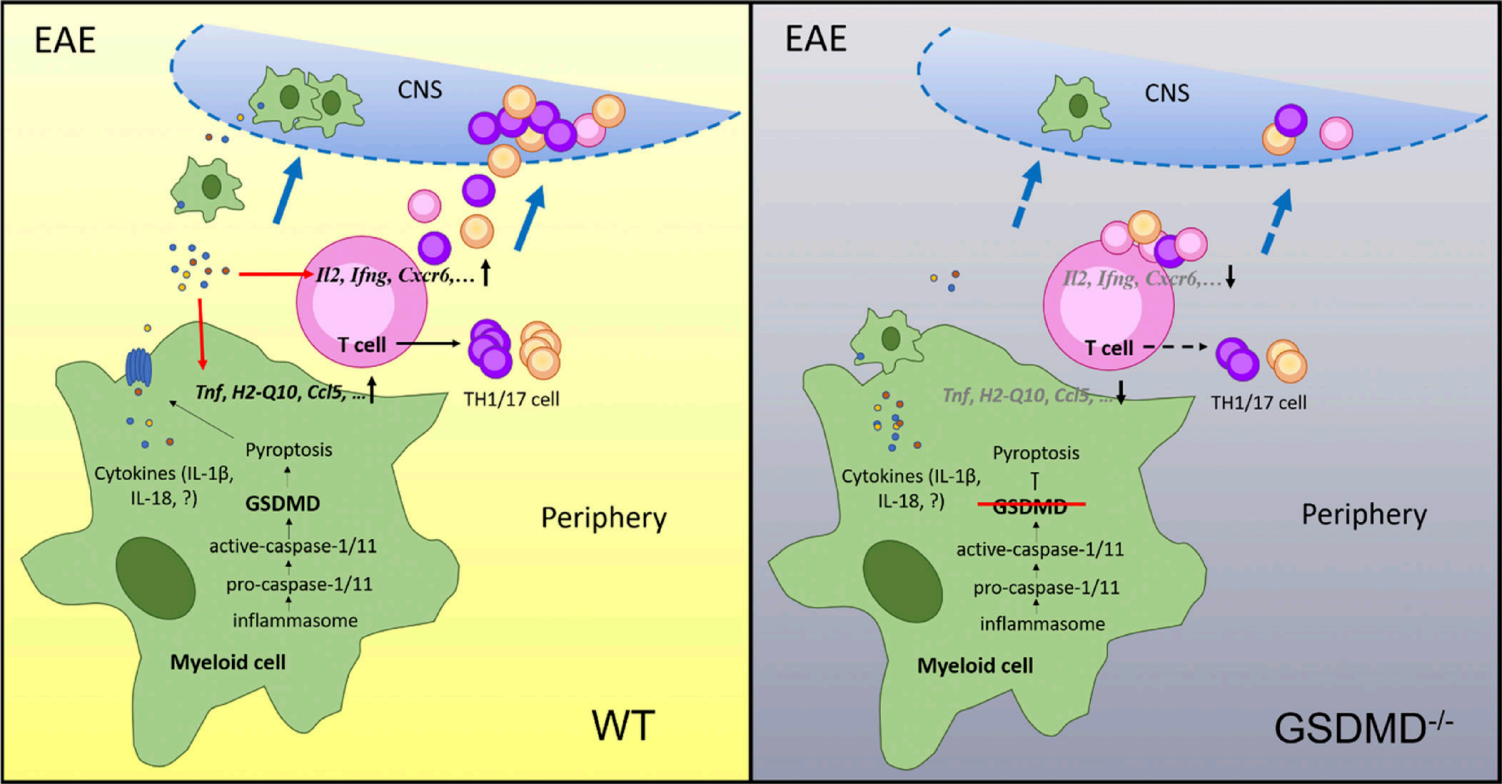
**Model for GSDMD function in EAE pathogenesis.** During EAE development, peripheral myeloid cell–intrinsic GSDMD mediates pyroptosis to instigate inflammation and promote the activation and differentiation of T cells in the secondary lymphoid organs, thus driving T cell–mediated neuroinflammation and demyelination in the CNS of EAE.

In summary, our study is the first to report the physiological role of GSDMD as a key mediator in autoimmune inflammation and pathogenesis during EAE. We propose a model in which peripheral myeloid cell–intrinsic GSDMD mediates pyroptosis to instigate inflammation and promote the activation and differentiation of T cells in the secondary lymphoid organs, thus driving T cell–mediated neuroinflammation and demyelination in the CNS of EAE (Fig. 10). The development of therapeutic strategies to specifically target GSDMD-mediated pyroptosis might be useful for the inhibition of CNS inflammation and MS treatment.

## Materials and methods

### Mice

Female mice with the C57BL/6 background were used in this study. The *Gsdmd*^−/−^ mice were kindly provided by Dr. Feng Shao (National Institute of Biological Sciences, Beijing, China). The *Gsdmd*^flox/flox^ mice was generated using conditional gene targeting methods by Cyagen Biosciences. To generate this mouse, the strategy of gene targeting and construction of the conditional targeting vector were established to delete the exon 3 genomic region of *Gsdmd* gene by flanking loxP sites and a Neo cassette. C57BL/6 mouse embryonic stem (ES) cells were electroporated with linearized *Gsdmd* targeting vector, and screening of the G418 resistant colonies was performed according to routine protocol. The homologous recombined ES cell clones were identified by PCR and confirmed by Southern blot analysis. The Neo selection cassette on the targeting construct was capable of removing itself after ES cell targeting without the need to breed to Flp deleter mice by the TetraOne ES Cell technique. Tail DNAs were genotyped using the primer sets specific to the floxed regions of *Gsdmd*^flox/+^ mice. Primers for 5′ floxed allele were as follows: primers P1 (5′-GGA​CCC​TGA​GAG​AAA​GAC​ATA​CCC​AT-3′) and P2 (5′-GTT​GGG​ATG​TTG​GGA​TGG​AAC​TCC-3′); WT mice results in generation of a 148-bp fragment with a 261-bp fragment corresponding to the floxed allele–positive mice. Primers for 3′ floxed allele were as follows: primers P3 (5′-CTC​TAC​TCC​TCT​GGT​CCT​ATT​TCC-3′) and P4 (5′- CAC​AGC​ACT​ACG​TTC​CAT​CGG​T-3′). The 364-bp fragment for the WT allele and the 420-bp fragment for the floxed allele were reconfirmed for heterozygous *Gsdmd* ^flox/+^ mice. *Gsdmd* floxed mice were crossed with *lysozyme M*-Cre mice (*LysM*-Cre; The Jackson Laboratory) to generate myeloid cell–conditional *Gsdmd* KO mice (*Gsdmd*^flox/flox^ *LysM*-Cre) or with *CD4*-Cre mice (The Jackson Laboratory) to produce T cell–conditional *Gsdmd* KO mice (*Gsdmd*^flox/flox^ CD4-Cre). For the microglia-conditional *Gsdmd* KO mice, we crossed *Gsdmd* floxed mice with *Cx3cr1*-CreERT2-EYFP mice (The Jackson Laboratory) to generate *Gsdmd*^flox/flox^ *Cx3cr1*-CreERT2-EYFP mice, and then the mice were i.p injected with 3 mg tamoxifen (T5648; Sigma-Aldrich) dissolved in 200 µl corn oil (C8267; Sigma-Aldrich) for 5 d consecutively to induce the expression of Cre recombinase. After 6 wk, the tamoxifen-induced mice were microglia–conditional *Gsdmd* KO mice and used for the EAE study. All mice were kept in a barrier facility, and all animal experiments were conducted in accordance with the procedure approved by the Ethical Review Committee for Laboratory Animal Welfare of Nanjing Medical University.

### Antibodies and reagents

Antibodies to GSDMD (ab219800, ab209845) and CD31 (ab24590) were from Abcam. Anti–caspase-11 (17D9, C1354), anti–α-tubulin (T9026), anti–β-actin (A1978), and anti-GFAP (G3893) were from Sigma-Aldrich. Anti–caspase-11 (NB120-10454) was from Novus. Anti–caspase-1 (AG-20B-0042-C100) was from Adipogen. Anti-Iba1 (019–19741) was from Wako. Anti-CD45-FITC (30-F11,11-0451-82), anti-CD8a-PE (53–6.7,12-0081-83), anti-CD11b-APC (M1/70,17-0112-82), anti-CD11b-FITC (M1/70,11-0112-82), anti-F4/80-APC (BM8,17-4801-82), anti-Gr-1-PerCP-Cyanine5.5 (RB6-8C5,45-5931-80), anti-CD11c-PE (N418,12-0114-81), anti-B220-PerCP-Cyanine5.5 (RA3-6B2,5-0452-80), anti-NK1.1-PE-Cyanine7 (PKB6,25-5941-82), anti-IL17-PE (eBio18B7,12-7177-81), anti-IFN-γ-PerCP-Cyanine5.5 (XMG1.2,85-45-7311-82), anti-Hu/MoCD44-FITC (IM7,11-0441-82), anti-Foxp3-APC (FJK-16s,17-5773-80A), anti-CD62L-APC (MEL-14,17-0621-82), anti-CD25-PE (PC61.5,12-0251-81B), anti-CD19-APC (eBio1D3,17-0193-82), and anti-CD3e-FITC (145-2C11,11-0031-81) were from eBioscience. Anti-CD4-APC-Cy7(GK1.5,100414), anti-Ki-67-PE (11F6,151209), anti-Cxcr6-Briliant Violet 421 (SA051D1,151109), anti-TNF-α-PE (MP6-XT22,506306), and anti-CCL5-PE (2E9/CCL5,149104) were from BioLegend. FAM-FLICA Caspase-1 Assay Kit (98) was from ImmunoChemistry. Anti-IL4 (11B11, 16–7041-85), anti–IFN-γ (RA-6A2,16-7312-85), anti-IL12/IL23p40 (C17.8,16-7123-81), mouse IL-6 recombinant protein (14–8061-62), mouse IL-12 p70 recombinant protein (14–8121-62), and mouse TGF β 1 recombinant protein (14–8342-62) were from Invitrogen. Pertussis toxin (180) was from List Biological Laboratories. *Mycobaterium tuberculosis* H37Ra (231141) was from BD. Incomplete Freund’s adjuvant (F5506) was from Sigma-Aldrich. MOG35-55 peptide (residues 35–55, Met-Glu-Val-Gly-Trp-Tyr-Arg-Ser-Pro-Phe-Ser-Arg-Val-Val-His-Leu-Tyr-Arg-Asn-Gly-Lys) was synthesized by Sangon Biotech.

### Induction and assessment of EAE

To induce EAE, MOG_35-55_ peptide (200 μg per mouse) was emulsified with CFA (50 μl per mouse, including 4 mg/ml *M*. *tuberculosis* H37Ra) and 50 μl incomplete Freund’s adjuvant. Pertussis toxin (250 ng per mouse) was applied intravenously on days 0 and 2 after immunization. Mice were assessed daily for clinical signs of EAE in a blinded fashion. EAE score was evaluated as follows: 0.5, partial tail paralysis; 1, tail paralysis; 1.5, reversible corrective reflex impairment; 2, corrective reflex impairment; 2.5, one hindlimb paralysis; 3, both hindlimbs paralysis; 3.5, both hindlimbs paralysis and one forelimb paralysis; 4, hindlimb and forelimb paralysis; and 5, death.

### Bone marrow chimeras

The recipient mice were subjected to lethal-dose irradiation (10 Gy), and 1 d later, bone marrow cells (10 × 10^6^) derived from the tibiae and femurs of donor mice were i.v. injected into lethally irradiated mice. Lethal dose irradiation can eliminate bone marrow and peripheral immune cells without affecting the radiation-resistant CNS resident cells in the recipient mice, so the peripheral immune system of chimeric mice will be reconstructed by bone marrow cells from donor mice. After 8 wk, the chimeric mice were then subjected to EAE induction.

### Adoptive transfer of CD4^+^ T cells and Th17 cells

CD4^+^ T cells were isolated from spleens of WT and *GSDMD*^−/−^ mice at 12 d after EAE induction by using CD4 negative selection MicroBeads (Stem Cell). Isolated T cells were adoptively transferred by i.v. injection (3 × 10^6^) to RAG-1 mice or i.c.v. injection (2 × 10^6^) to WT mice. Mice were also i.v. injected with pertussis toxin on days 0 and 2 after transfer. Polarized Th17 cells were also used to induce passive EAE. We first collected DLN cells from WT and *GSDMD*^−/−^ donor mice at 12 d after MOG_35-55_ immunization, and cultured cells for 5 d with MOG_35-55_ (25 µg/ml) and IL-23 (20 ng/ml) under Th17 cell polarizing conditions. The MOG_35-55_-specific polarized Th17 cells were then adoptively transferred by i.v. injection (3 × 10^7^) to sublethal irradiated (5 Gy) WT recipient mice or *GSDMD*^fl/fl^ and *GSDMD*^fl/fl^*LysM*-Cre mice.

### In vitro T cell proliferation and Th1/Th17 cell polarization

CD4^+^ T cells were isolated from DLNs of WT mice at 12 d after EAE induction by CD4 negative selection MicroBeads (Stem Cell). The isolated T cells (1 × 10^6^) were cocultured with WT or *GSDMD*^−/−^ DCs (2 × 10^5^) that were pretreated with LPS (1 µg/ml) for 4 h and washed twice before co-culture. For proliferation experiments, T cells were labeled with 5 µM CFSE and cocultured with DCs in the presence of MOG_35-55_ (3 µg/ml). For polarization experiments, T cells were cocultured with DCs in the presence of MOG_35-55_ (3 µg/ml) under Th1 cell polarizing conditions (anti–IL-4, 10 µg/ml; IL-12, 10 ng/ml) or Th17 cell polarizing conditions (anti–IL-4, 10 µg/ml; anti–IL-12, 10 µg/ml; anti–IFN-γ, 10 µg/ml; IL-6, 50 ng/ml; and TGF-β, 5 ng/ml). CFSE dilution and Th1/Th17-cell percentages were determined by FACS at 5 d after co-culture.

### Histological and immunohistochemistry

All spinal cord tissue sections used here were 5 µm thick. For paraffin-embedded tissue, spinal cords collected from PBS-perfused mice were fixed in 4% paraformaldehyde overnight. Sections were stained with H&E or with LFB for evaluation of inflammation and demyelination, respectively. For immunohistochemical staining, sections were blocked and incubated with primary antibodies and horseradish peroxidase-conjugated secondary antibodies after heat-induced antigen retrieval. Diaminobenzidine was used for detection. Images were captured with a Nikon 50i microscope.

### Immunofluorescence staining

Tissue sections were incubated at 4°C overnight with primary antibodies to IBA1, GFAP, MBP, and CD4. Slides were then incubated with indicated secondary antibodies. The nuclei were counterstained with DAPI (Sigma-Aldrich). Slides were dried and mounted using ProLong Antifade mounting medium (Beyotime Biotechnology). Slides were visualized using a Nikon 50i fluorescent microscope.

### Flow cytometry

For preparation of immune cells in the CNS, brains, and spinal cords from MOG_35–55_-immunized mice were excised and digested at 37°C with collagenase type IV (0.5 mg/ml; Sigma-Aldrich) and DNase I (10 U/ml; Roche) in RPMI 1640 under agitation (200 rpm) conditions for 60 min. The digested tissues were filtered through a 100-µm filter, and the plunger end of the syringe was used to push the cells over the filter. Homogeneous cell suspensions were centrifuged over the Percoll density gradient (GE Healthcare) and separated by collecting the interface fractions between 37% and 70% Percoll. Mononuclear cells were isolated from the interface. The cells were suspended in PBS containing 2% (wt/vol) FBS. After intensive washing, cells were stained with fluorochrome-conjugated surface marker antibodies for FACS analysis. The following antibodies were used: CD45, CD4, CD8, and CD11b. For intracellular cytokine staining, cells were stimulated with phorbol 12-myristate 13-acetate (Multi Sciences), ionomycin (Multi Sciences), and brefeldin A (Invitrogen) for the 4 h of culture. Cells were fixed and permeabilized with the Intracelluar Fixation & Permeabilization Buffer Set (eBioscience) and then subjected to cytokine staining flow cytometry analyses. All flow cytometry was performed on an Attune NxT flow cytometer (Thermo Fisher Scientific), and data were analyzed by FlowJo 7.6.1 software.

### Quantitative RT-PCR

Total RNA was extracted by using TRIzol reagent (Life) and subjected to cDNA synthesis. Quantitative RT-PCR was performed using SYBR Green Supermix (Vazyme). The expression of a single gene was calculated by a standard curve method and standardized to the expression of *Hprt*.

The following primers were used: *Ccl22* S As, 5′-TCG​GTT​CTT​GAC​GGT​TAT-3′ and 5′-TGC​TGC​CAG​GAC​TAC​ATC-3′; *Ccl5* S As, 5′-GAC​ACC​ACT​CCC​TGC​TGC​TT-3′ and 5′-ACA​CTT​GGC​GGT​TCC​TTC​G-3′; *Ccr4* S As, 5′-TCC​AAA​CAG​ACC​CAA​CAA-3′ and 5′-ACC​ACC​CAG​GAT​GAA​ACT-3′; *Ccr6* S As, 5′-TCA​CGA​CTC​GGA​TTG​CTC-3′ and 5′-CTG​CTG​GGT​ATG​GGA​CTG-3′; *Cxcr3* S As, 5′-GTT​GGC​TGA​TAG​GTA​GAT​GAA-3′ and 5′-CTG​CTG​TCC​AGT​GGG​TTT-3′; *Cxcr6* S As, 5′-CAC​CCA​AAT​GAG​CAA​GCA​AA-3′ and 5′-ATG​GAT​GAT​GGG​CAT​CAA​GAG​TCA​G-3′; *Ifnγ* S As, 5′-CAT​GAG​TAT​TGC​CAA​GTT​TGA​GG-3′ and 5′-CGA​CTC​CTT​TTC​CGC​TTC​C-3′; *Itga6* S As, 5′-GTT​CTG​TTC​GCC​CTC​TGC-3′ 5′-TGC​CTG​CTC​TAC​CTG​TCC-3′; *Il1b* S As, 5′-ATG​CCA​CCT​TTT​GAC​AGT​GAT​G-3′ and 5′-GTT​GAT​GTG​CTG​CTG​CGA​GA-3′; *cxcl1* S As, 5′-AGC​TGC​GCT​GTC​AGT​GCC​T-3′ and 5′-TGT​GGC​TAT​GAC​TTC​GGT​TTG​G-3′; *Tnfa* S As, 5′-TAC​TGA​ACT​TCG​GGG​TGA​TCG-3′ and 5′-TCC​TCC​ACT​TGG​TGG​TTT​GC-3′; *Osm* S As, 5′-GAG​CCA​TCG​TCC​CAT​TCC-3′ and 5′-CTC​ACG​GTC​CAC​TAC​AAC​AC-3′; *H2-q10* S As, 5′-AGT​ATT​GGG​AGC​GGG​AGA-3′ and 5′-CCG​TCG​TAT​GCG​TAT​TGC-3′; *H2-ob* S As, 5′-GAC​AAC​AGT​AAT​GCT​GGA​AAT​GA-3′ and 5′-TGA​GCC​TTG​AGA​TGG​ATA​ACA​AC-3′; *Cd3e* S As, 5′-TGG​AGC​AAG​AAT​AGG​AAG​GC-3′ and 5′-GGT​TGG​GAA​CAG​GTG​GTG-3′; *Il2* S As, 5′-CTC​TGC​GGC​ATG​TTC​TGG​A-3′ and 5′-TCA​TCA​TCG​AAT​TGG​CAC​TCA-3′; *Hprt* S As, 5′-GTC​CCA​GCG​TCG​TGA​TTA​GC-3′ and 5′-TGG​CCT​CCC​ATC​TCC​TTC​A-3′.

### Immunoblot

Spinal cords from MOG_35–55_-immunized mice were excised and placed in NP-40 lysis buffer (50 mM Tris-HCl, pH 7.4, containing 150 mM NaCl, 1% [vol/vol] Igepal, 10% [wt/vol] glycerol, 50 mM NaF, 1 mM Na_3_VO_4_, 1 mM dithiothreitol, 1 mM phenylmethylsulphonyl fluoride, and complete protease inhibitor “cocktail” [Sigma-Aldrich]), followed by tissue homogenization and then incubation for 30 min at 4°C. The lysates were centrifuged for 10 min at 14,000 *g* for removal of cell debris and nuclei. Supernatants were assayed for protein concentration, and lysate samples (50 µg) were resolved by SDS-PAGE, transferred to nitrocellulose membranes, and analyzed by immunoblot with the appropriate antibodies. Immunoreactivity was visualized by the Odyssey Imaging System (LI-COR Biosciences) or enhanced chemiluminescence.

### RNA-seq analysis

For RNA-seq, CD11b^+^ and CD4^+^-T cells were isolated from spleens of WT and *GSDMD*^−/−^ mice at 14 d after immunization by using CD11b and CD4 MicroBeads (Stem Cell), respectively. RNA isolation, library construction, and sequencing were performed on a BGISEQ-500 (Beijing Genomic Institution). Clean reads were mapped to the mouse genome (GRCm38.p5) by HISAT2. For gene expression analysis, the matched reads were calculated and then normalized to FPKM. Fold changes were calculated for all possible comparisons, and a 1.2-fold cutoff was used to select genes with expression changes. KEGG pathway analysis was performed using the R package, using significantly differentially expressed genes (P < 0.05) as target genes. Raw data files and processed files have been deposited in the Gene Expression Omnibus under accession no. GSE126289).

### rIL-1β, rIL-18, and disulfiram treatment in EAE mice

rIL-1β (25 ng per mouse; Stem Cell) and rIL-18 (25 ng per mouse; R&D Systems) were i.v. injected into WT and GSDMD^−/−^ mice on days 4, 8, 12, and 16 (where day 0 is MOG_35–55_ immunization). Disulfiram (10 mg/kg) was i.p. injected into WT mice daily after immunization. Clinical signs and histological analysis were performed as described above.

### Statistical analyses

The data were analyzed by GraphPadPrism 7.0 software and are presented as the mean ± SEM. The statistics were analyzed by using an unpaired *t* test for two groups and multiple *t* test or two-way ANOVA for multiple groups. P values were provided as *, P < 0.05; **, P < 0.01; and ***, P < 0.001.

### Data availability

Sequencing data were deposited in the Gene Expression Omnibus under accession no. GSE126289.

### Online supplemental material

Fig. S1 shows the activation of GSDMD and caspase-11 in DLNs and spinal cord and that GSDMD deficiency inhibits the activation of macrophages, microglia, and astrocytes in CNS of EAE mice. Fig. S2 shows GSDMD conditional KO mice strategy. Fig. S3 shows that GSDMD deficiency does not affect the development of T and B cells. Fig. S4 shows that GSDMD deficiency does not affect the development of myeloid cells and NK cells. Fig. S5 shows that GSDMD deficiency in myeloid cells does not affect the development of lymphocytic cells and myeloid cells.

## Supplementary Material

Supplemental Materials (PDF)
