# Supplementary material for: Gasdermin D in peripheral myeloid cells drives neuroinflammation in experimental autoimmune encephalomyelitis
Source: J Exp Med. 2019 Aug 29;216(11):2562–81. doi: 10.1084/jem.20190377 (PMC6829591; doi:10.1084/jem.20190377)
Supplement: Supplemental Materials (PDF) [file JEM_20190377_FigS1-S5.pdf]

Supplemental material

Li et al., https://doi.org/10.1084/jem.20190377

Supplement Figure.1

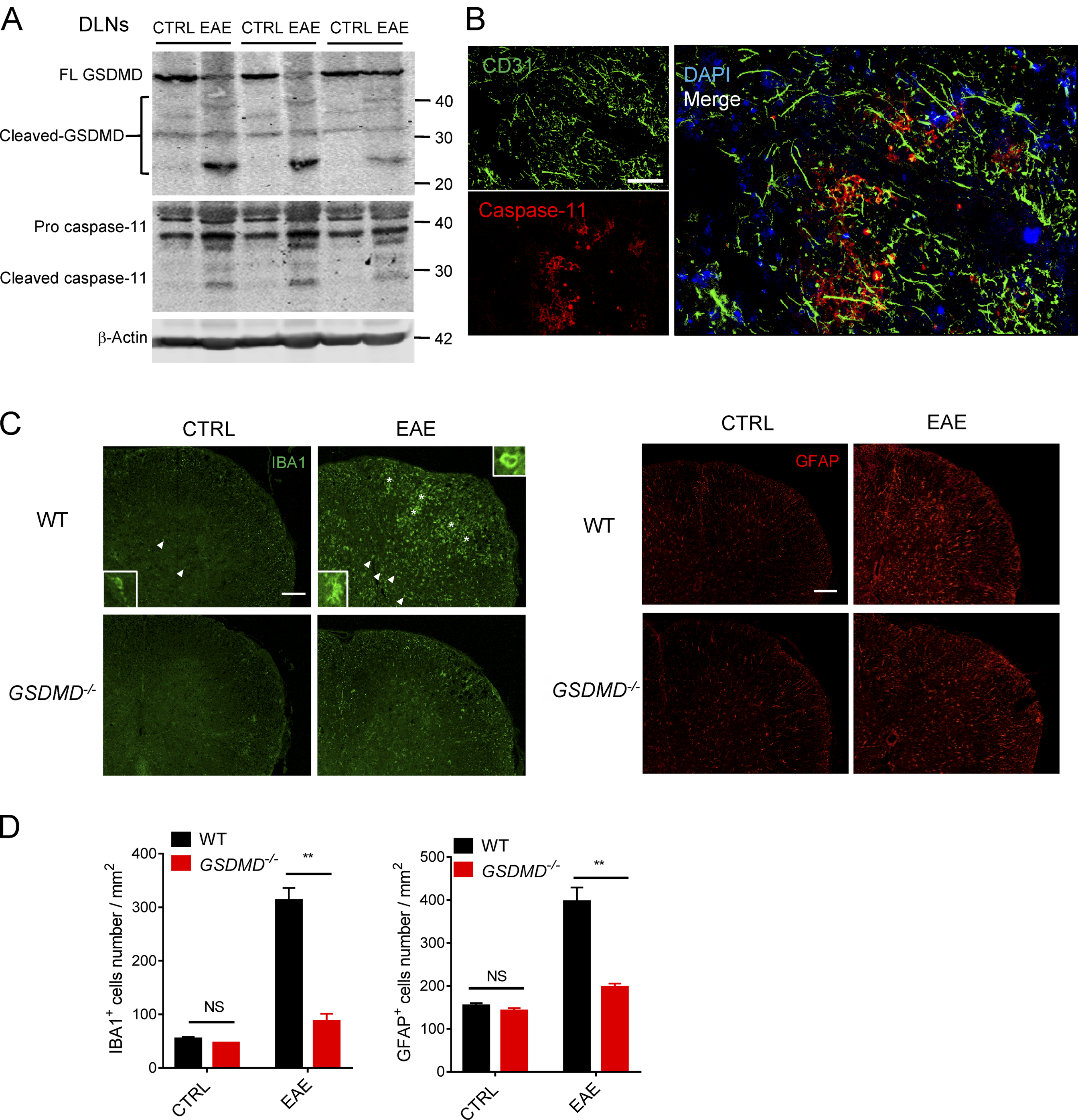

Figure S1. **The expression of GSDMD and caspase-11 in DLNs and spinal cord, and the activation of macrophages, microglia, and astrocytes during EAE are suppressed in *GSDMD*$^{-/-}$ mice. (A)** Immunoblot analysis of full-length (FL) and cleaved GSDMD and caspase-11 in the DLNs from three pairs of control (CTRL) or EAE-induced WT mice at day 18 after immunization. **(B)** Immunofluorescent labeling of CD31 (green) and caspase-11 (red) shows capillaries and caspase-11 expression in the spinal cord of EAE-induced WT mice at day 18 after immunization. DAPI staining of nuclei is also included. Scale bar, 100 µm. **(C and D)** Immunofluorescent analysis of IBA1 (green) and GFAP (red) positive cells in the spinal cords of EAE-induced WT and *GSDMD*$^{-/-}$ mice at day 18 after immunization. Image in left shows macrophages infiltrating near the edge of spinal cord (indicated by asterisk) and microglia in the center of spinal cord (indicated by arrowheads) with different morphology. Scale bars, 100 µm. Representative images (C) and quantified cell numbers (D; n = 3 mice per group) are shown, respectively. Data are representative of three independent experiments. **, $P < 0.01$. Error bars show means ± SEM. Multiple unpaired t test for C and D.

Supplement Figure.2

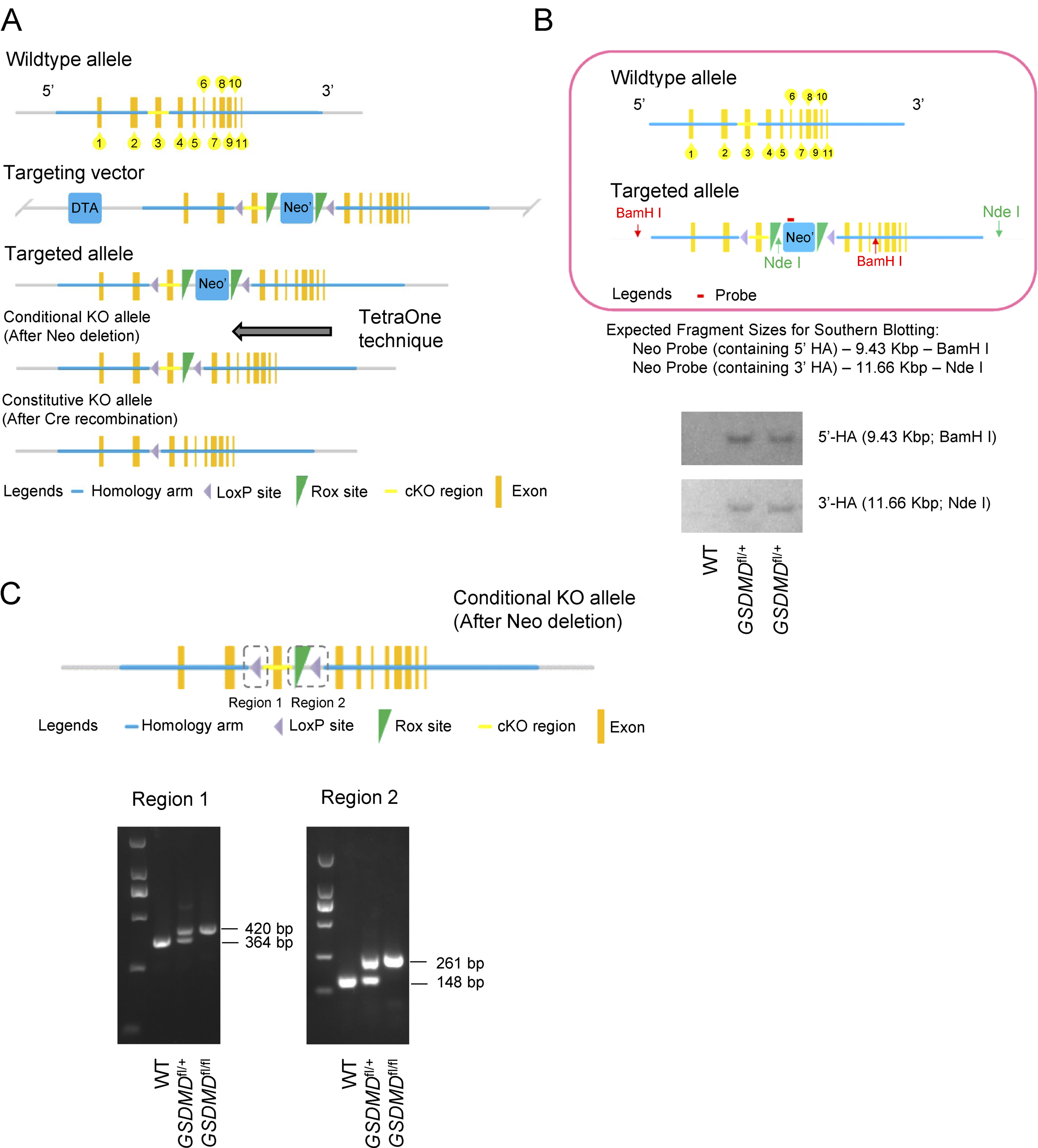

Figure S2. **GSDMD conditional KO mice strategy. (A)** Targeting vector design for generation of a mouse strain with GSDMD exon 3 flanked by loxp sites. The Neo selection cassette can be removed itself after ES cell targeting by TetraOne technique without the need to breed to Flp deleter mice. **(B)** Regions selected for Southern blotting and the result of Southern blotting analysis. **(C)** Genotyping strategy and representative image for genotyping analysis of GSDMD loxp sites.

Supplement Figure.3

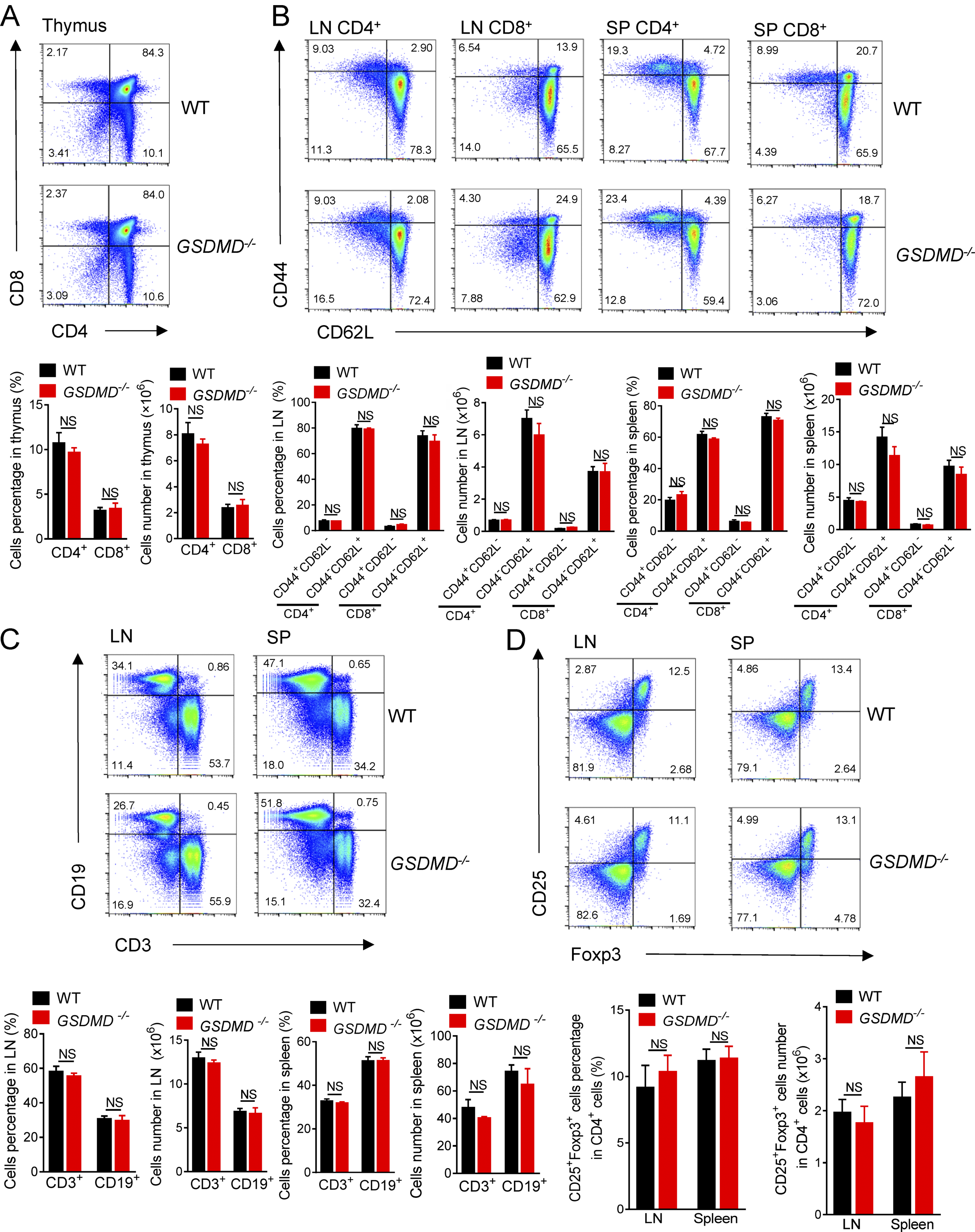

Figure S3. **The development of T and B cells is not impaired in *GSDMD*$^{-/-}$ mice. (A)** Flow-cytometric analysis of CD4^{+} and CD8^{+} T cells in thymus from 6-mo-old WT and *GSDMD*$^{-/-}$ mice (n = 4 or 5 mice per group). **(B)** Flow-cytometric analysis of CD62L^{+} naive T cells and CD44^{+} effect T cells in LN and spleen (SP) from 6-mo-old WT and *GSDMD*$^{-/-}$ mice (n = 4 mice per group). **(C and D)** Flow-cytometric analysis of CD3^{+} T cell, CD19^{+} B cell (C), and Foxp3^{+}CD25^{+} T reg cells (D) in LN and spleen from 6-mo-old WTs and *GSDMD*$^{-/-}$ mice (n = 4 or 5 mice per group). Data are presented as a representative plot and summary graph of the percentages. Error bars show means ± SEM. Multiple unpaired *t* test for A–D.

Supplement Figure.4 -1

A

Bone marrow

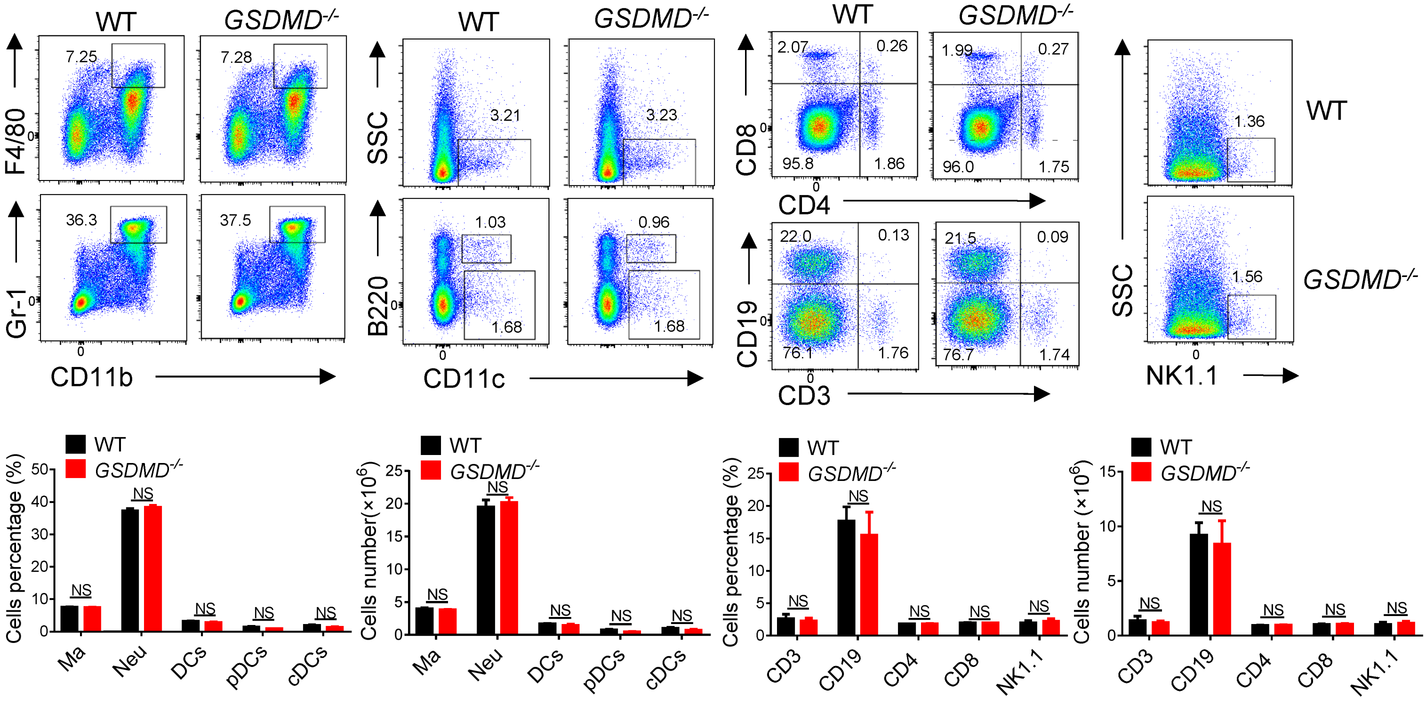

B

Blood

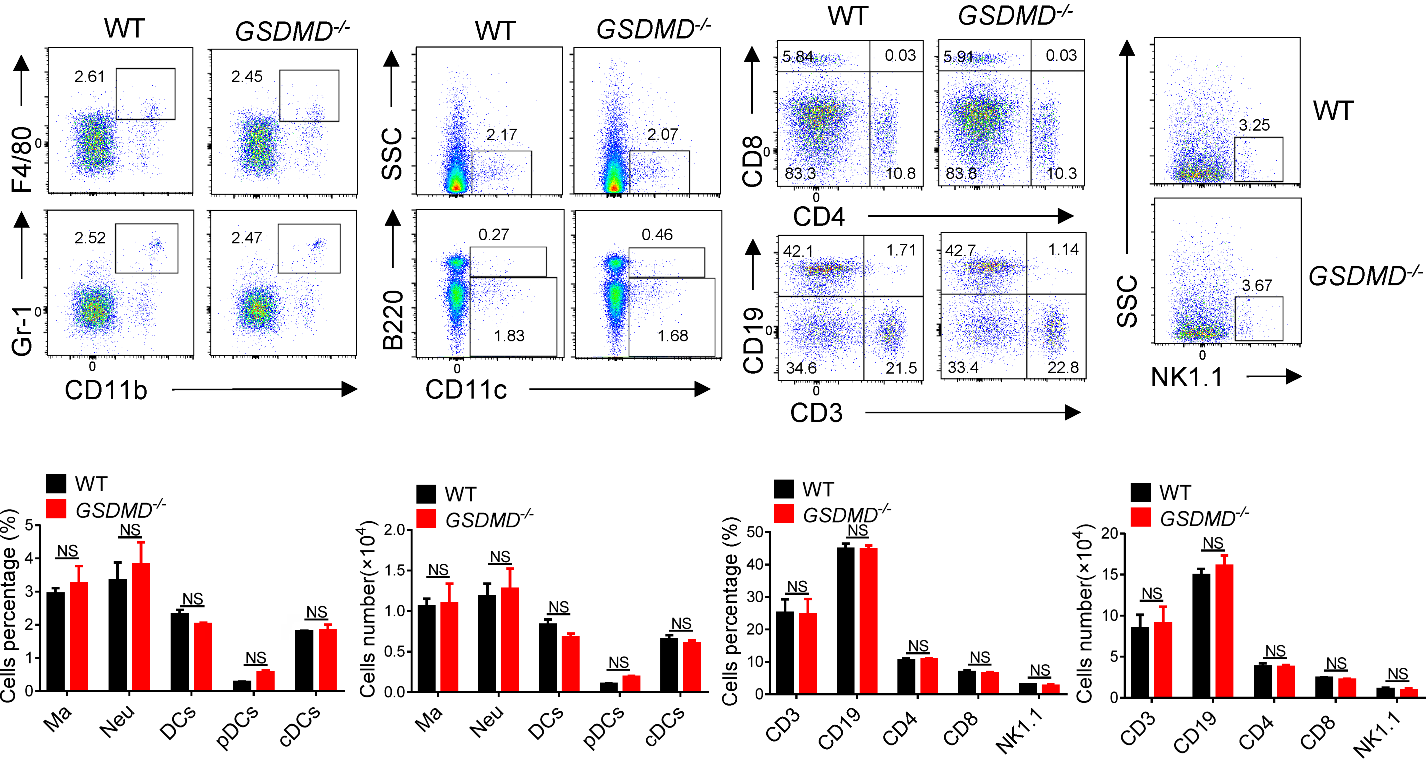

Supplement Figure.4 -2

C

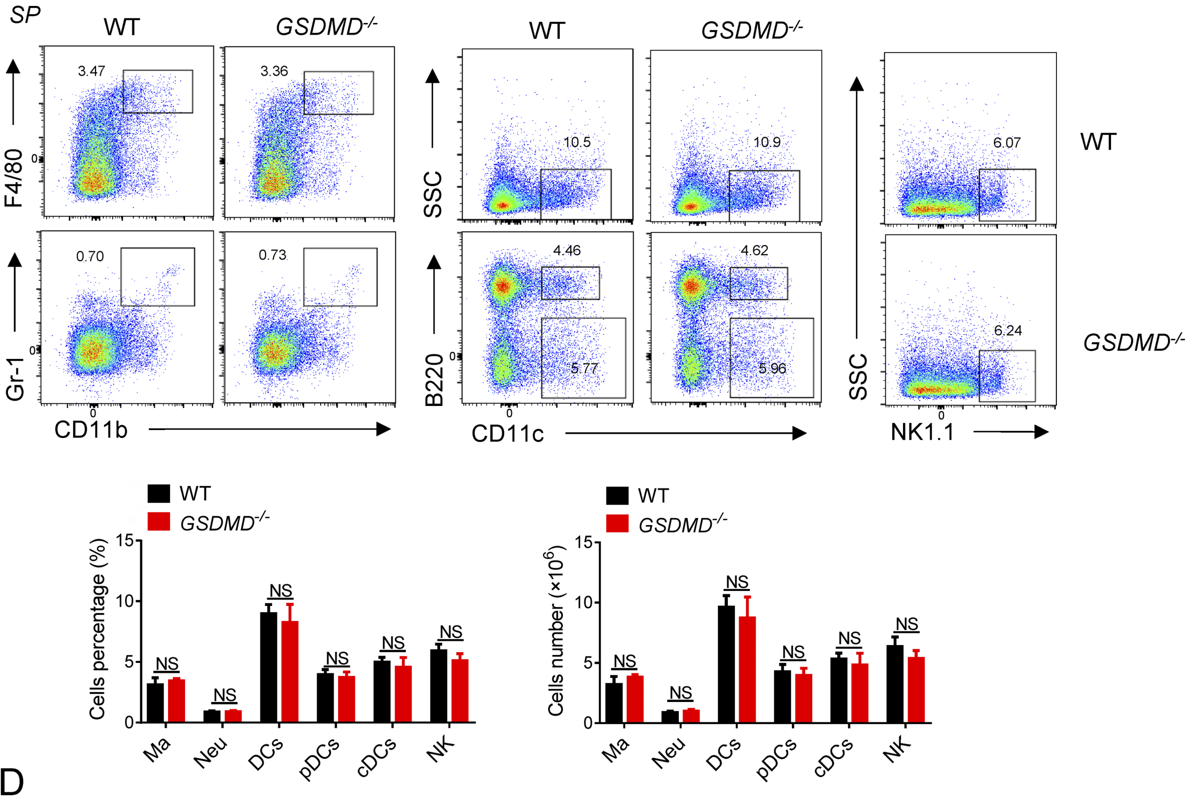

D

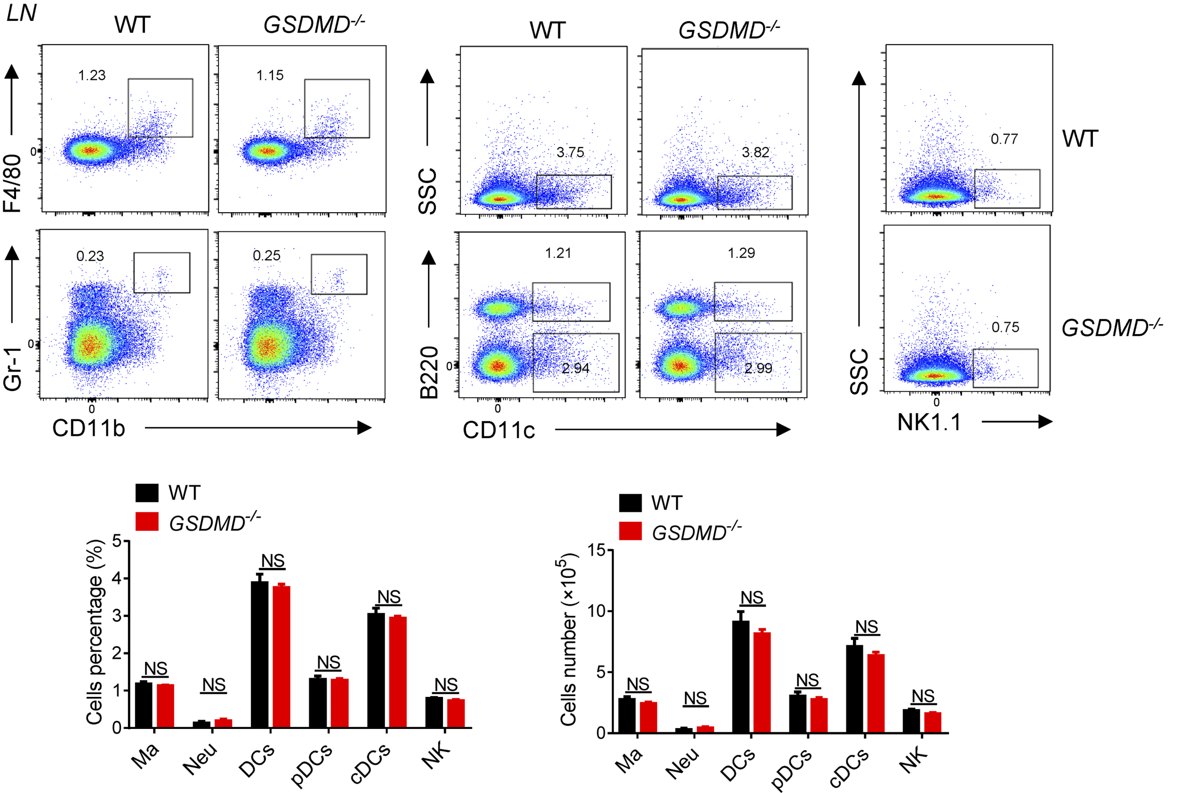

Figure S4. **The development of myeloid cells is not impaired in *GSDMD*$^{-/-}$ mice. (A and B)** Flow-cytometric analysis of macrophages (Ma; CD11b^{+}F4/80^{+}), neutrophils (Neu; CD11b^{+}Gr-1^{+}), DCs (CD11c^{+}, including cDCs [CD11c^{+}B220^{-}] and pDCs [CD11c^{+}B220^{+}]), T cells (including CD3^{+}, CD4^{+}, and CD8^{+}), B cells (CD19^{+}), and NK cells (NK1.1^{+}) in bone marrow (A) and blood (B) from 8-wk-old WT and *GSDMD*$^{-/-}$ mice (n = 3 mice per group). **(C and D)** Flow-cytometric analysis of macrophages (CD11b^{+}F4/80^{+}), neutrophils (CD11b^{+}Gr-1^{+}), DCs (CD11c^{+}, including cDCs [CD11c^{+}B220^{-}] and pDCs [CD11c^{+} B220^{+}]) and NK cells (NK1.1^{+}) in spleen (SP; C) and LN (D) from WT and *GSDMD*$^{-/-}$ mice (n = 3 mice per group). Data are presented as a representative plot and summary graph of the percentages and absolute cell numbers. Error bars show means ± SEM. Multiple unpaired t test for A–D. SSC, side scatter.

Supplement Figure.5 -1

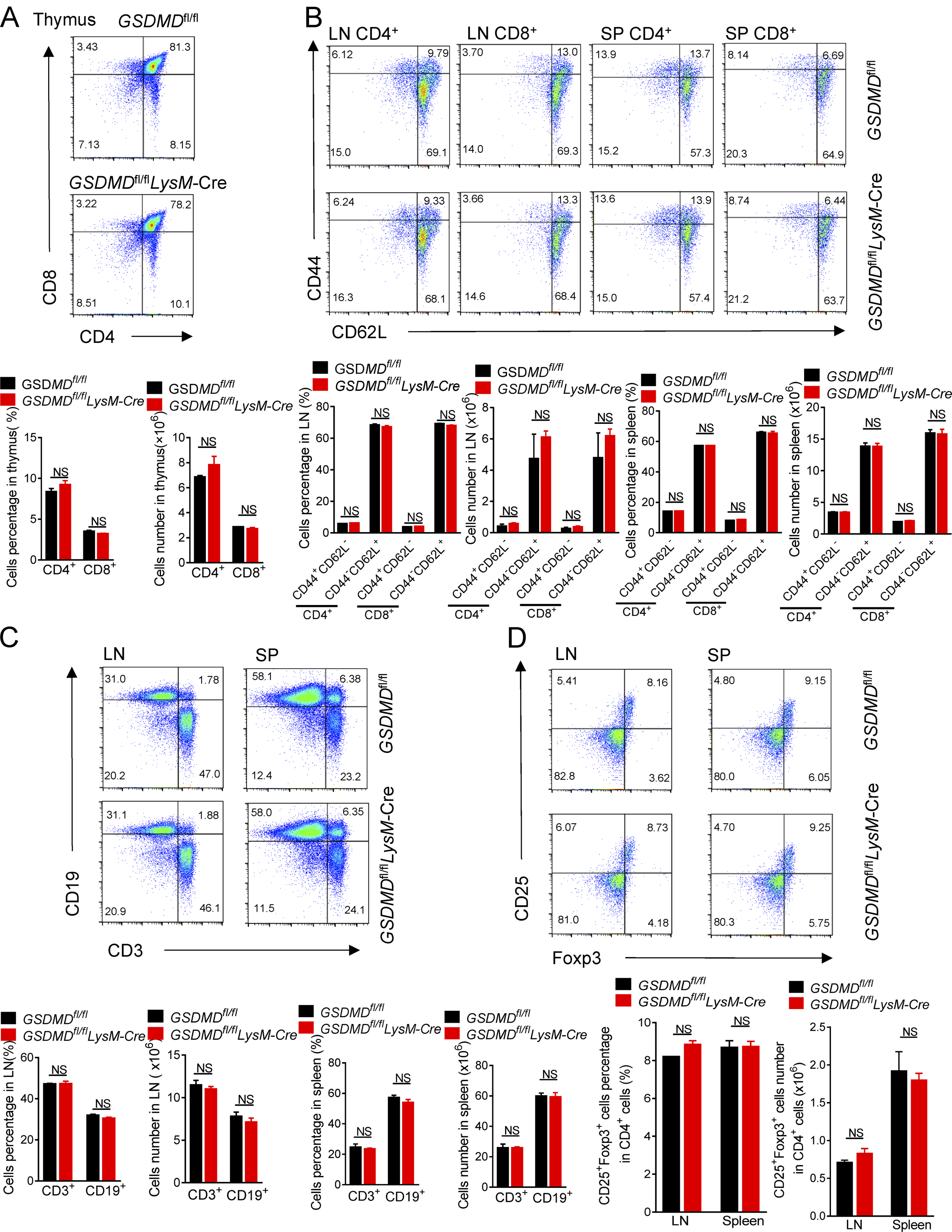

Supplement Figure.5 -2

E

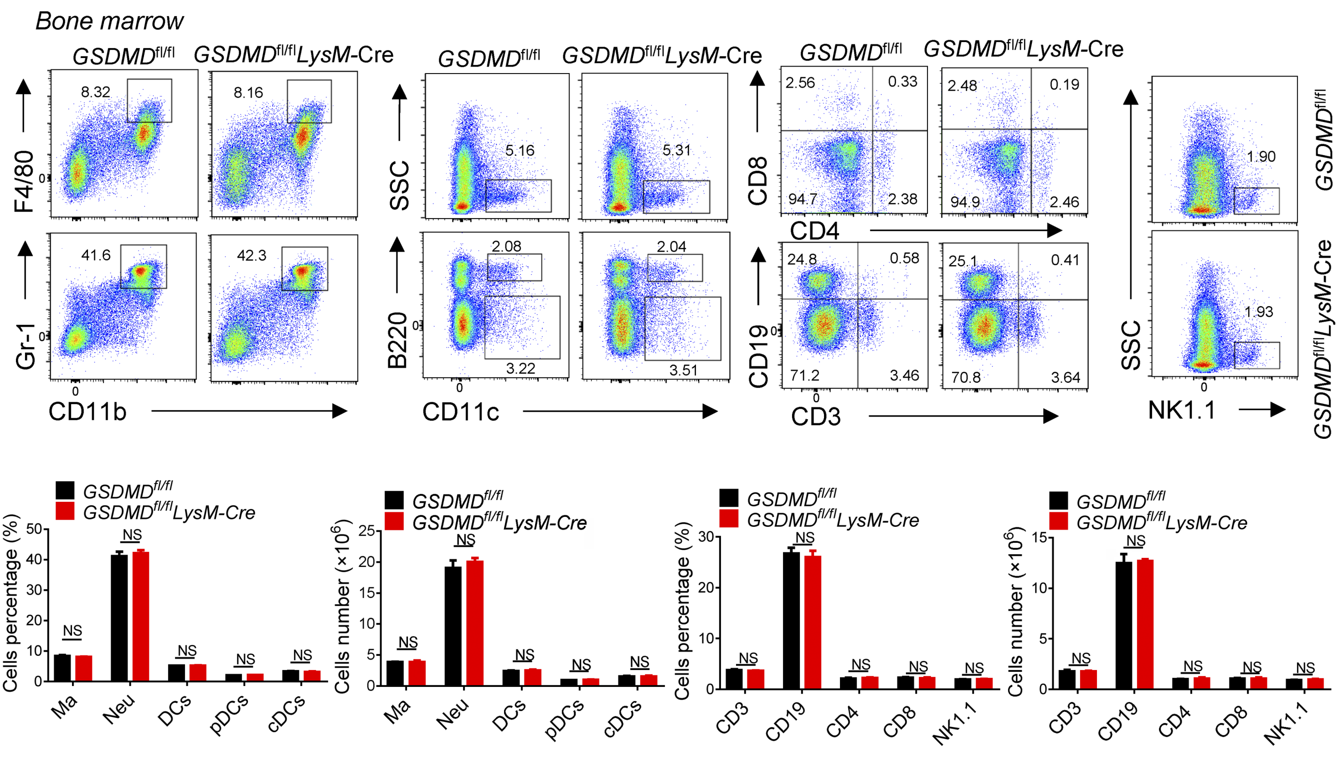

F

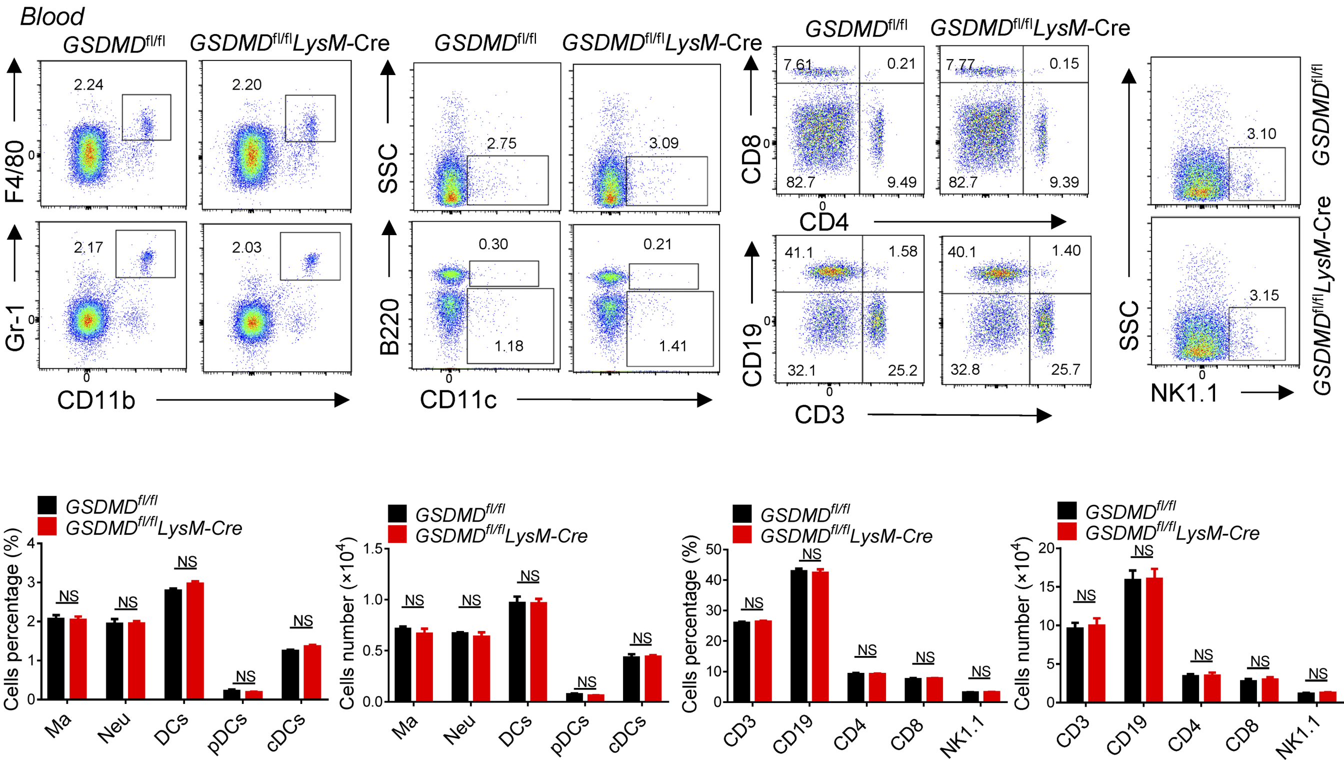

Supplement Figure.5 -3

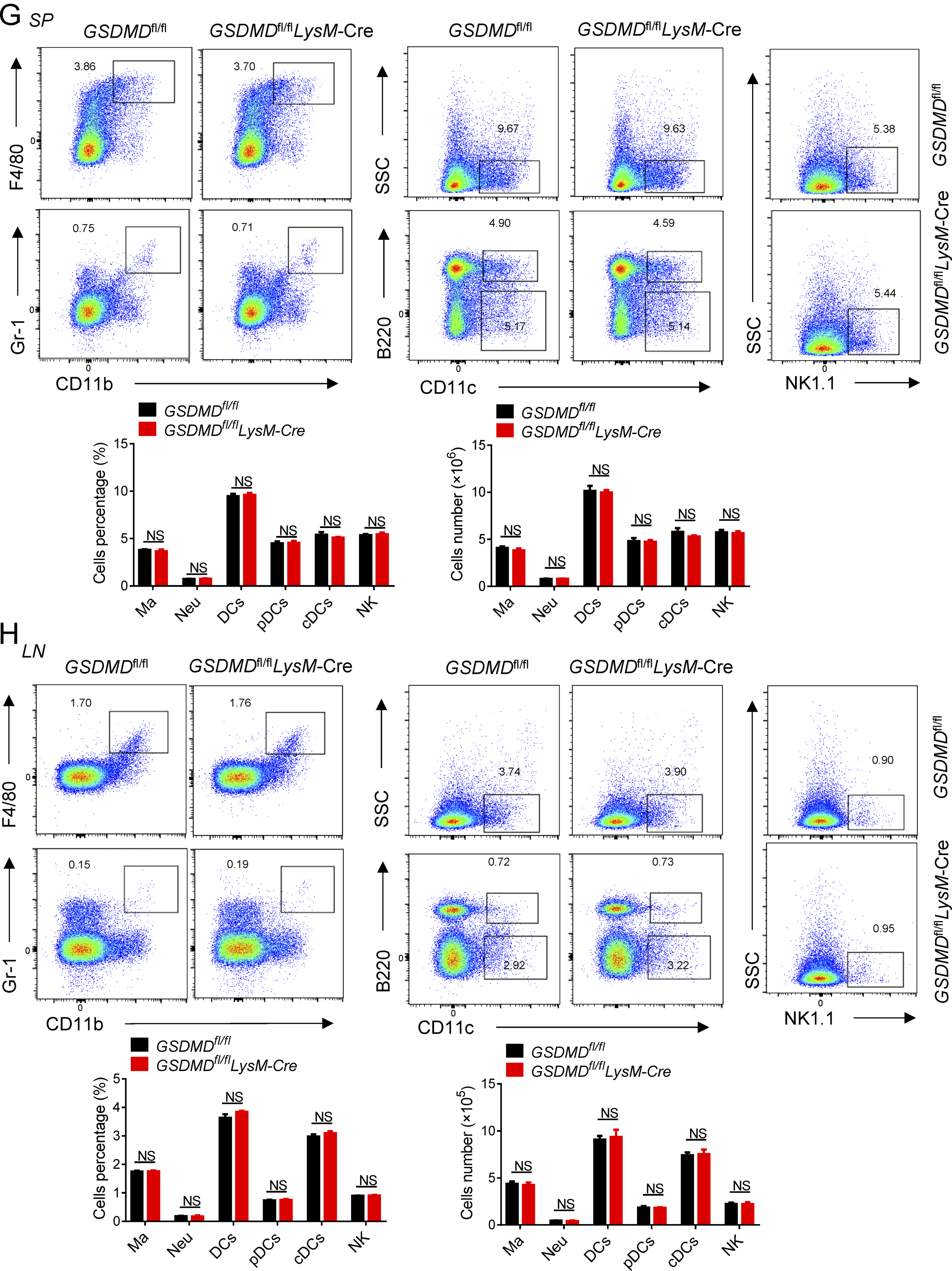

Figure S5. **The development of lymphocytic cells and myeloid cells is not impaired in *GSDMD*$^{fl/fl}$*LysM*-Cre mice. (A)** Flow-cytometric analysis of CD4$^+$ and CD8$^+$ T cells in thymus from 8-wk-old *GSDMD*$^{fl/fl}$*LysM*-Cre and littermate control *GSDMD*$^{fl/fl}$ mice (n = 3 mice per group). **(B)** Flow-cytometric analysis of CD62L$^+$ naive T cells and CD44$^+$ effect T cells in LN and spleen from 8-wk-old *GSDMD*$^{fl/fl}$*LysM*-Cre and littermate control *GSDMD*$^{fl/fl}$ mice (n = 3 mice per group). **(C and D)** Flow-cytometric analysis of CD3$^+$ T cell, CD19$^+$ B cell (C), and Foxp3$^+$CD25$^+$ T reg cells (D) in LN and spleen (SP) from 8-wk-old *GSDMD*$^{fl/fl}$*LysM*-Cre and littermate control *GSDMD*$^{fl/fl}$ mice (n = 3 mice per group). **(E and F)** Flow-cytometric analysis of macrophages (Ma; CD11b$^+$F4/80$^+$), neutrophils (Neu; CD11b$^+$Gr-1$^+$), DCs (CD11c$^+$, including cDCs [CD11c$^+$B220$^-$] and pDCs [CD11c$^+$B220$^+$]), T cells (including CD3$^+$, CD4$^+$, and CD8$^+$), B cells (CD19$^+$), and NK cells (NK1.1$^+$) in bone marroow (E) and blood (F) from 8-wk-old *GSDMD*$^{fl/fl}$*LysM*-Cre and littermate control *GSDMD*$^{fl/fl}$ mice (n = 3 mice per group). **(G and H)** Flow-cytometric analysis of macrophages (CD11b$^+$F4/80$^+$), neutrophils (CD11b$^+$Gr-1$^+$), DCs (CD11c$^+$, including cDCs [CD11c$^+$ B220$^-$] and pDCs [CD11c$^+$ B220$^+$]) and NK cells (NK1.1$^+$) in spleen (G) and LN (H) from 8-wk-old *GSDMD*$^{fl/fl}$*LysM*-Cre and littermate *GSDMD*$^{fl/fl}$ mice (n = 3 mice per group). Data are presented as a representative plot, summary graph of the percentages, and absolute cell numbers. Error bars show means ± SEM. Multiple unpaired t test for A–H. SSC, side scatter.
